# Novel azo-benzoyl acetone metal complexes for inhibition of atmospheric steel corrosion

**DOI:** 10.1038/s41598-026-64011-y

**Published:** 2026-08-07

**Authors:** Yusif S. El-Sayed, Nadia El-Wakiel, Kamal El-Baradie, Basma M. Salem

**Affiliations:** Chemistry Department, Faculty of Science, Tanta University, Tanta, 31527 Egypt

**Keywords:** Benzoyl acetone, Metal complexes, TGA, Spectral studies, X-ray diffraction, Rust, Atmospheric corrosion, Steel corrosion, Materials Studio software, Chemistry, Materials science

## Abstract

The atmospheric corrosion threatens the structural reliability and service life of a wide range of engineering systems, which can cause economically significant degradation phenomena affecting metallic materials in industrial environments. Therefore, a New 2-((1H-indazol-5-yl) diazenyl)-1-phenylbutane-1,3-dione ligand (L) and its divalent cobalt, nickel, and copper complexes were synthesized and characterized. By using Elemental Analysis (CHN), Inductively Coupled Plasma (ICP), Molar Conductance (Λm), X-ray Powder Diffraction (XRD), Thermogravimetric analysis (TGA), and magnetic moment measurements. Also, Fourier Transform Infrared (FT-IR), Proton Nuclear Magnetic Resonance (^1^H-NMR), Carbon Nuclear Magnetic Resonance (^13^C-NMR), mass, and Ultraviolet-Visible (UV-Vis) spectroscopy. Through the nitrogen atom of the azo group and one oxygen atom of the carbonyl group, the ligand performs the function of a bidentate ligand. From the characterization and theoretical results, it was found that Co(II) and Ni(II) have an octahedral geometry, whereas the Cu(II) complex has a tetrahedral structure. The activity of ligand and its metal complexes as inhibitors to reduce the rate or prevent atmospheric corrosion of iron was investigated. Materials Studio 2017 software was used to study the adsorption on the Fe(110) surface. The computed adsorption energies follow the trend Cu(II) complex > Ni(II) complex > L > Co(II) complex, which is qualitatively consistent with the experimentally observed inhibition efficiency order. Scanning Electron Microscope (SEM) analysis proved that the specimen covered with Cu(II) complex has a good inhibitory effect that slows down the rate of corrosion compared with the blank and untreated specimen.

**Supplementary Information:**

The online version contains supplementary material available at 10.1038/s41598-026-64011-y.

## Introduction

Azo compounds exhibit exceptional stability arising from the conjugation of the (-N = N-) group with aromatic ring systems, making them among the most significant classes of organic colorants^1,2^. Azo dyes based on heterocyclic scaffolds are particularly valued for their intense color properties and for their ability to coordinate with transition metals through the azo group’s strong donor characteristics^3–5^. When combined with a β-diketone moiety in a single molecular framework, the resulting azo β-diketone ligands offer dual functionality, which includes chelating capacity and enhanced complex stability^6^. Metal chelates of azo dye have demonstrated broad utility across industries, cosmetics, corrosion inhibition, and catalysis^7–12^. Atmospheric corrosion of steel, in particular, represents a persistent industrial challenge, as the exposure of metallic structures to ambient conditions leads to significant economic losses^13,14^. Organic inhibitors are among the most practical and effective strategies for mitigating this corrosion, acting by adsorbing onto the metal surface to form a protective film that limits the atmospheric attack^15,16^. The inhibition efficiency of such compounds is governed by their electronic structures, specifically the presence of heteroatoms (N, P, S, O) and π electron systems that facilitate coordination with the empty d-orbitals of iron atoms^17,18^. Inhibitors bearing multiple heteroatoms or conjugated systems provide superior protection owing to the presence of more adsorption sites^17^. Despite considerable research on organic corrosion inhibitors^18–22^, atmospheric corrosion, as opposed to solution-phase corrosion, has received comparatively less attention, and the inhibitory potential of azo β-diketone complexes incorporating indazole derivatives is underexplored^23^. Previous studies reported that indazole derivatives have a high adsorption capacity on the metal surface^24^. Also, β-diketone complexes show influence on the mild steel corrosion^25,26^. DFT is regarded as an effective computational technique for analyzing the electronic structure and characteristics of molecules, particularly transition metal complexes^27–30^. The SEM and EDX provide good information on the surface morphology of the steel in the presence and absence of the inhibitor^11^. A literature survey revealed a lack of azo β-diketone ligands derived from 5-aminoindazole and their Co(II), Ni(II), and Cu(II) complexes as inhibitors for atmospheric steel corrosion.

In this work, we discuss the synthesis and characterization of an azo dye ligand based on 5-aminoindazole with benzoyl acetone, and its divalent cobalt, nickel, and copper complexes. Different analytical and spectral techniques were used to confirm the structures. The primary study of atmospheric corrosion to show the capability of the synthesized compounds to inhibit the steel corrosion in the atmosphere, utilizing the weight loss technique, was investigated. The inhibition behavior of the synthesized compounds was further investigated by computational studies using the adsorption locator of the Material Studio software.

## Experimental

### Chemicals and instrumentations

5-aminoindazole (97%), benzoyl acetone (99%), Cobalt(II) chloride hexahydrate (98%), Nickel(II) chloride hexahydrate (99.9%), and Copper(II) chloride (99%), dimethyl sulfoxide (DMSO, 99.9%), triethylamine (99%), and ethanol (95%) were obtained purchased from Sigma-Aldrich and used as received. The instruments used have been described in our previous work cited in^31^.

### Synthesis of the investigated compounds

#### Synthesis of 2-((1 H-indazol-5-yl) diazenyl)-1-phenylbutane-1,3-dione (azo dye ligand (L))

**Scheme 1:**
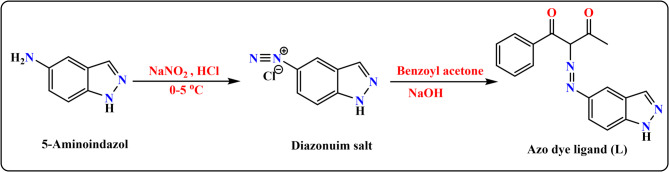
Synthesis pathway of azo-dye ligand (L).

Diazonium Salt was synthesised by dissolving 3.99 g (0.03 mol) of 5-aminoindazole in 50.0 mL of dist.H_2_O and 4.00 mL of concentrated HCl, and then it was cooled to (0–5) ^o^C. To this mixture, a cold aqueous NaNO_2_ solution 2.06 g (0.03 mol) in 15.0 mL of dist.H_2_O was added dropwise at (0–5) ^o^C and stirred for 25 min. This diazonium salt was added to a solution that contained 4.86 g (0.03 mol) of benzoyl acetone dissolved in 25.0 mL of NaOH (10%). The produced reddish-brown azo dye ligand **(L)**, which was separated out, was kept in an ice bath under stirring for 30 min at 0 °C and 30 min at room temperature. The precipitate was filtered off, washed with dist. H_2_O. Finally dried over anhydrous calcium chloride in a vacuum desiccator. The melting point of the obtained azo dye ligand was 120 °C. The synthesis pathway is shown in Scheme 1.

#### Synthesis of metal complexes

An ethanolic solution of hydrated metal chlorides 0.004 mol [0.951 g CoCl_2_.6H_2_O, 0.950 g NiCl_2_.6H_2_O, and 0.537 g CuCl_2_], 0.004 mol (1.224 g) of the azo dye ligand, and a few drops of triethylamine was refluxed for 24 h. The solid complexes obtained were filtered, washed with ethanol, and dried over anhydrous CaCl_2_ in a desiccator. All of these metal complexes decompose with a melting point above 300 °C.

### Theoretical studies

All quantum chemical calculations were carried out using the Gaussian 09 W software. Full geometry optimizations (FOPT) were performed for all studied compounds. The restricted B3LYP functional (RB3LYP) was applied for the azo-dye ligand, which was treated as a closed-shell singlet system (multiplicity = 1), using the 6-311^++^G (d, p) basis set. For the transition metal complexes, unrestricted calculations (UB3LYP) were employed to account for the open-shell nature of the metal centers. The LanL2MB effective core potential basis set was used for Cu(II) and Ni(II) complexes with spin multiplicities of 2 (doublet) and 3 (triplet), respectively. The LanL2DZ effective core potential basis set was applied for the Co(II) complex with a spin multiplicity of 4 (quartet). All calculations were performed in the gas phase without solvation corrections, representative of atmospheric corrosion conditions. All compounds were treated as neutral species (charge = 0). Frontier Molecular Orbital (FMO) analysis, Molecular Electrostatic Potential (MEP) maps, and global reactivity descriptors were derived from the optimized geometries.

### Primary studies of atmospheric corrosion

#### Weight loss study

A series of steel sheets with dimensions of 2 × 1 × 0.2 cm was mechanically press-cut into coupons. Each sample had a tiny hole punched at its edge to allow suspension during exposure. Various grades of emery paper were used to polish the sheet surface until the mirror finish was achieved. An iron sheet of 99.92 weight% purity was used to prepare the Fe specimens. Before use, all specimens were cleaned using distilled water followed by acetone. Then, allowed to dry completely at room temperature. Inhibitor solutions (ligand and its complexes) were prepared at a concentration of 10^− 3^ M dimethyl sulfoxide (DMSO). The treated samples, which were fully immersed in the inhibitor solutions for 10 days at room temperature, began on 11 March. The treated specimens and the control sample were exposed to the atmosphere during the period from 21 March to 18 May (a total exposure period of 58 days). The atmospheric conditions were characterized by a temperature of 20–35 °C, relative humidity of 46–55%, negligible rainfall, and an urban atmosphere with significant dust and particulate deposition. Visual inspection of all specimens was carried out throughout the exposure period, and corrosion-induced changes were documented photographically at the following intervals: 0, 9, 11, 13, 16, 23, 32, 47, and 58 days. After 58 days exposure period, each specimen was cleaned to remove corrosion products. The weight loss was measured using an analytical balance with a precision of (± 0.0001 g). Each sample was weighed three times; the obtained values of weight loss represent the average of the three measurements to ensure accuracy and reliability.

#### Molecular Dynamics (MD) simulations

The interaction between the inhibitor molecules and the iron surface was investigated using molecular dynamics simulations. The adsorption locator module in Material Studio 2017 was used to simulate the compounds’ adsorption on the Fe(110) surface. The Fe(110) surface was selected because it represents the most stable and densely packed plane of iron. All of the system’s component structures were optimized using the Forcite Module^32^. A simulation box was used to simulate the interactions between the molecules involved and the Fe(110) surface with lattice parameters (a = 28.49 Å, b = 28.49 Å, c = 48.84 Å, α = 89.58°, β = 89.85°, and γ = 90.01°). A 15 × 20 supercell was created by extending the unit cell in both the x and y directions. Ultimately, the iron surface was covered with a vacuum slab that was 26 ˚A thick.

### SEM and EDX analysis

The scanning electron microscopy (SEM) and Energy Dispersive X-ray (EDX) spectra were applied to study the surface morphology of the iron surface^33^.

## Results and discussion

### Structural characterization

#### Elemental microanalysis and molar conductivity

All the synthesized compounds are solid, colored, stable towards air and moisture, and insoluble in most organic solvents except DMSO. The complexes’ analytical results validate the formation of 1:1 (M: L) for all complexes and are in agreement with the suggested molecular formula. Table 1. At room temperature, the metal complexes’ molar conductances in (10^− 3^M) DMSO fall between 7.51 and 2.30 ∧^− 1^ cm^− 2^ mol^− 1^. It is evident from the low molar conductance values that the metal complexes are not electrolytic^34^.

**Table 1:** Physical properties of metal complexes.

Compounds/molecular formula	Color	Mol. wt (practical)	Yield %	Elemental analysis%				Λm*
				C	H		M	
Azo-dye Ligand (L)	Reddish brown	306.32 (306.79)	92.8	66.66 (66.90)	4.61 (4.54)	18.29 (18.54)	–	–
Co(II) complex [Co L Cl_2_ (H_2_O)_2_]⋅1.5H_2_O	Coffee	499.21 (498.59)	89.54	40.90 (41.13)	4.24 (4.49)	11.22 (11.36)	11.81 (11.96)	2.30
Ni(II) complex [Ni L Cl_2_ (H_2_O)_2_]⋅2.5H_2_O	Brown	516.99 (516.92)	55.87	39.49 (39.73)	4.48 (4.69)	10.84 (10.72)	11.35 (10.78)	7.51
Cu(II) complex [Cu L Cl_2_]⋅H_2_O	Medium brown	458.5 (459.47)	58.94	42.82 (42.77)	3.81 (3.68)	11.75 (11.67)	13.33 (13.36)	2.42

*Molar conductance (∧^−1^ cm^2^ mol^−1^).

#### Mass spectroscopy

**Scheme 2:**
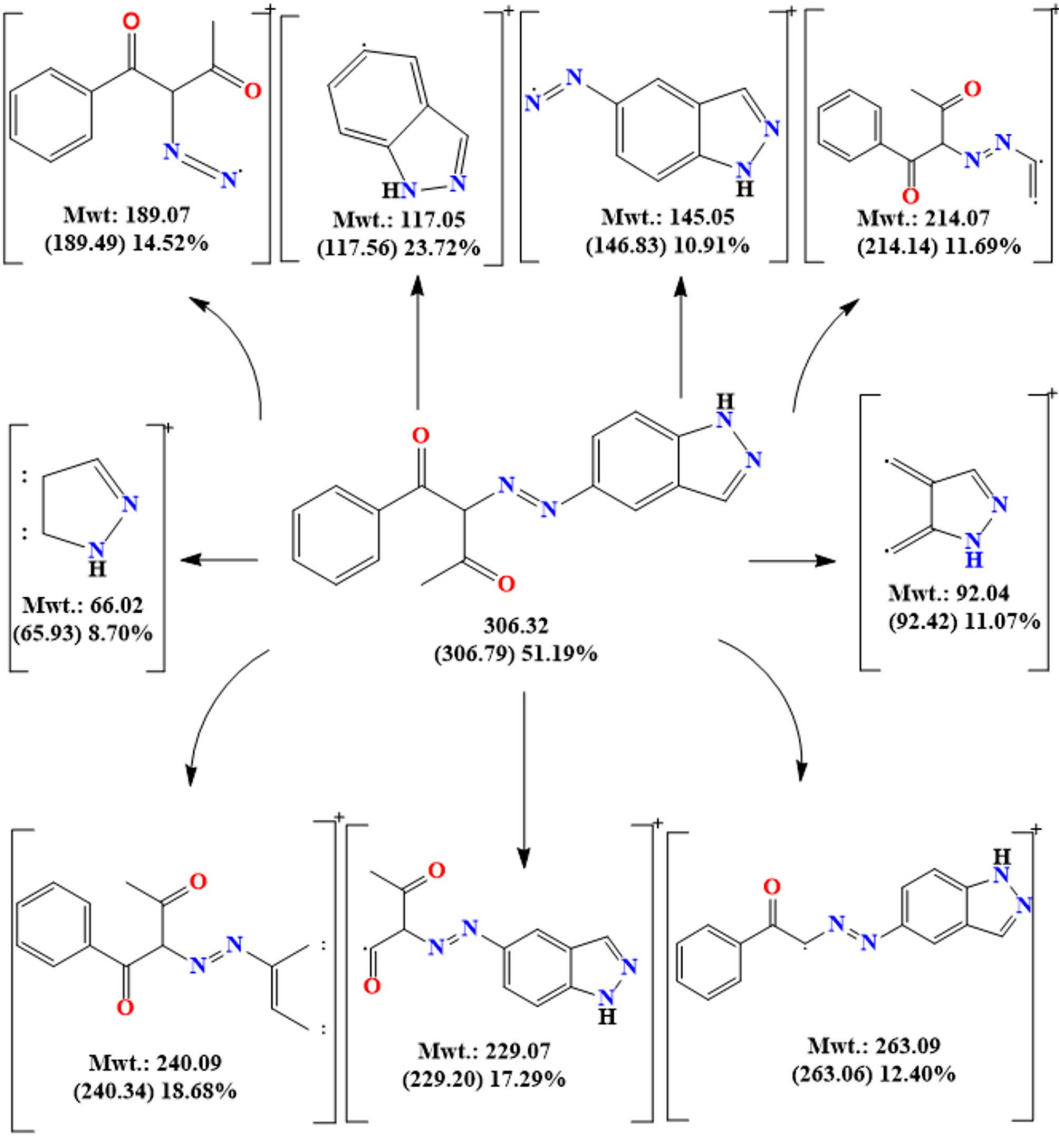
Fragmentation pathways of the ligand.

**Fig. 1:**
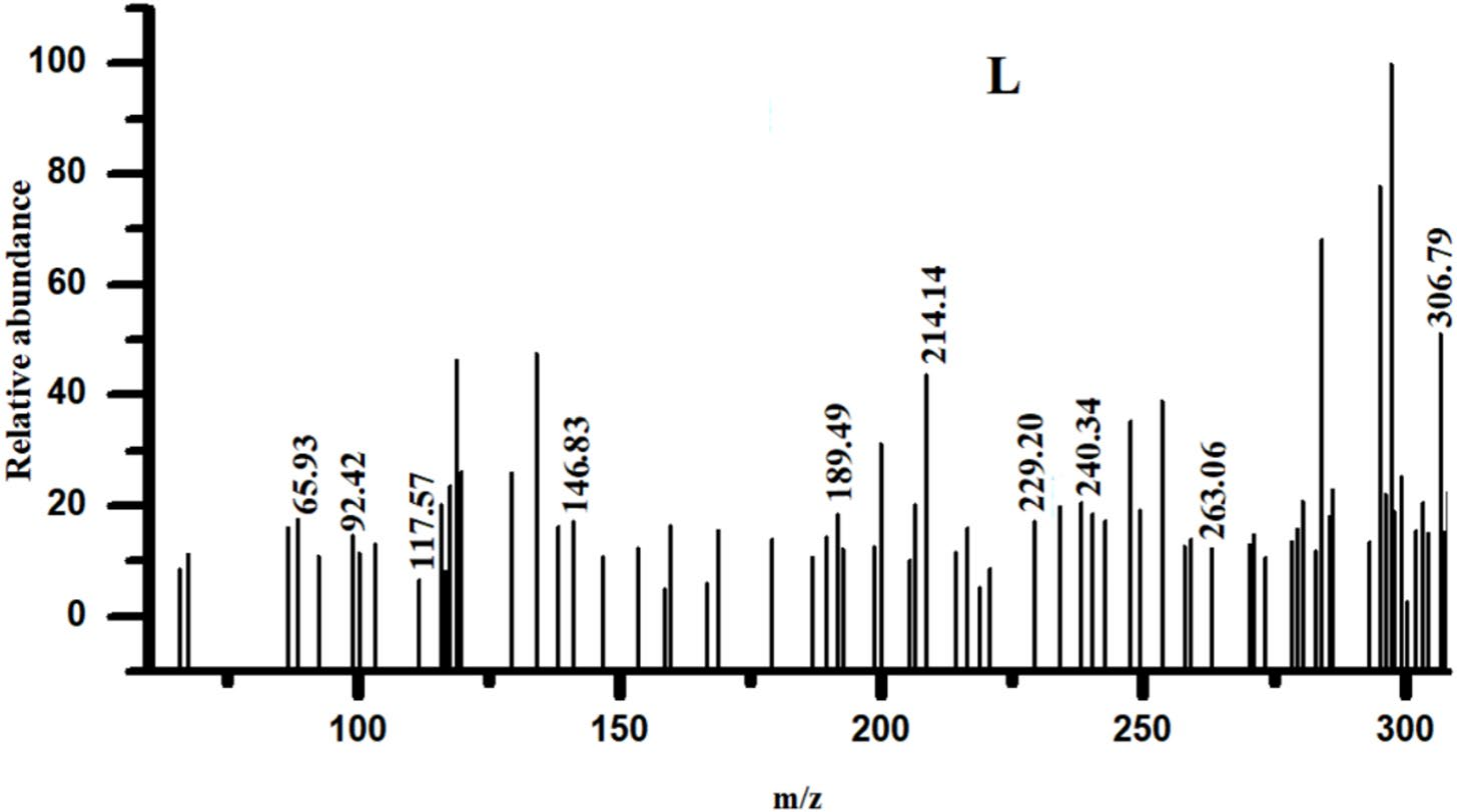
EI-Mass spectrum of Azo-dye ligand.

The ligand’s mass spectrum displayed a molecular ion peak at m/z 306.79, as shown in Fig. 1. It corresponds to the ligand’s molecular weight. The peaks at m/z 236.06, 240.32, 229.20, 214.14, 189.49, 146.83, 117.56, 92.42, and 65.93 are due to the fragments that are displayed in Scheme 2. The peaks at m/z = 189.49 and 146.83 prove that the azo compound has formed. The spectra of Co(II), Ni(II), and Cu(II) metal complexes are in Figures (S1- S3). Showed accurate molecular ion peaks at m/z 498.59, 516.92, and 475.93, respectively, which belong to the parent ion. The Co(II) complex’s mass spectrum showed peaks at m/z 498.59, 473.06, 437.01, 365.00, 307.94 and 74.94 that are corresponding to [Co(L)Cl_2_ (H_2_O)_2_]0.1.5H_2_O, [Co(L)Cl_2_ (H_2_O)_2_] (loss of the lattice water molecules), [Co(L)Cl_2_] (loss of lattice water and coordinated molecules) [Co(L)] (loss of lattice and coordinated water molecules and coordinated chloride ions), [L]^+^ and CoO, respectively. The results of both elemental analyses and mass spectroscopy of the prepared compounds are in agreement with each other. Verifying the suggested molecular formula for these complexes.

#### NMR spectra for the azo dye ligand (L)

**Fig. 2:**
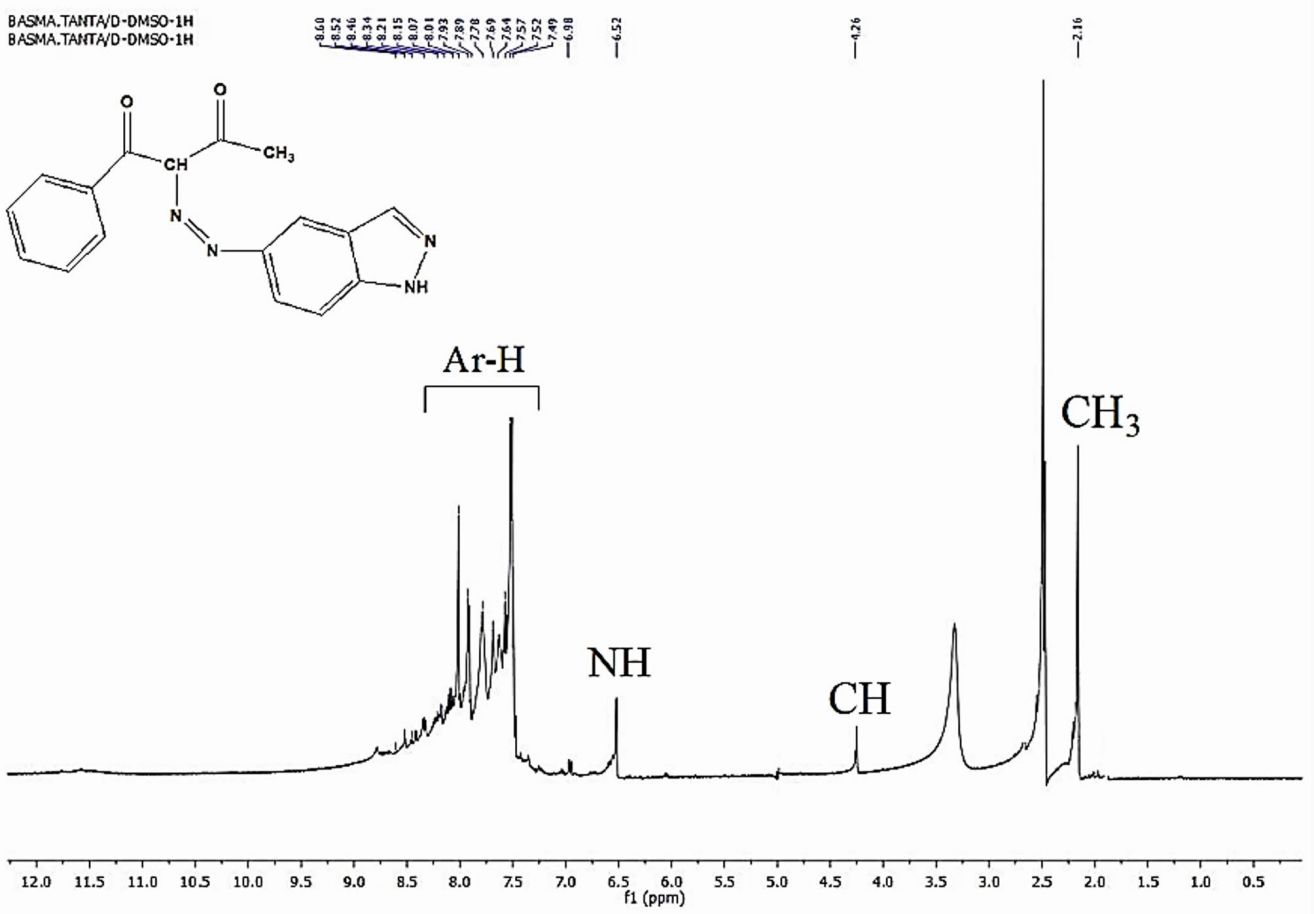
^1^H-NMR spectrum of the Ligand.

**Fig. 3:**
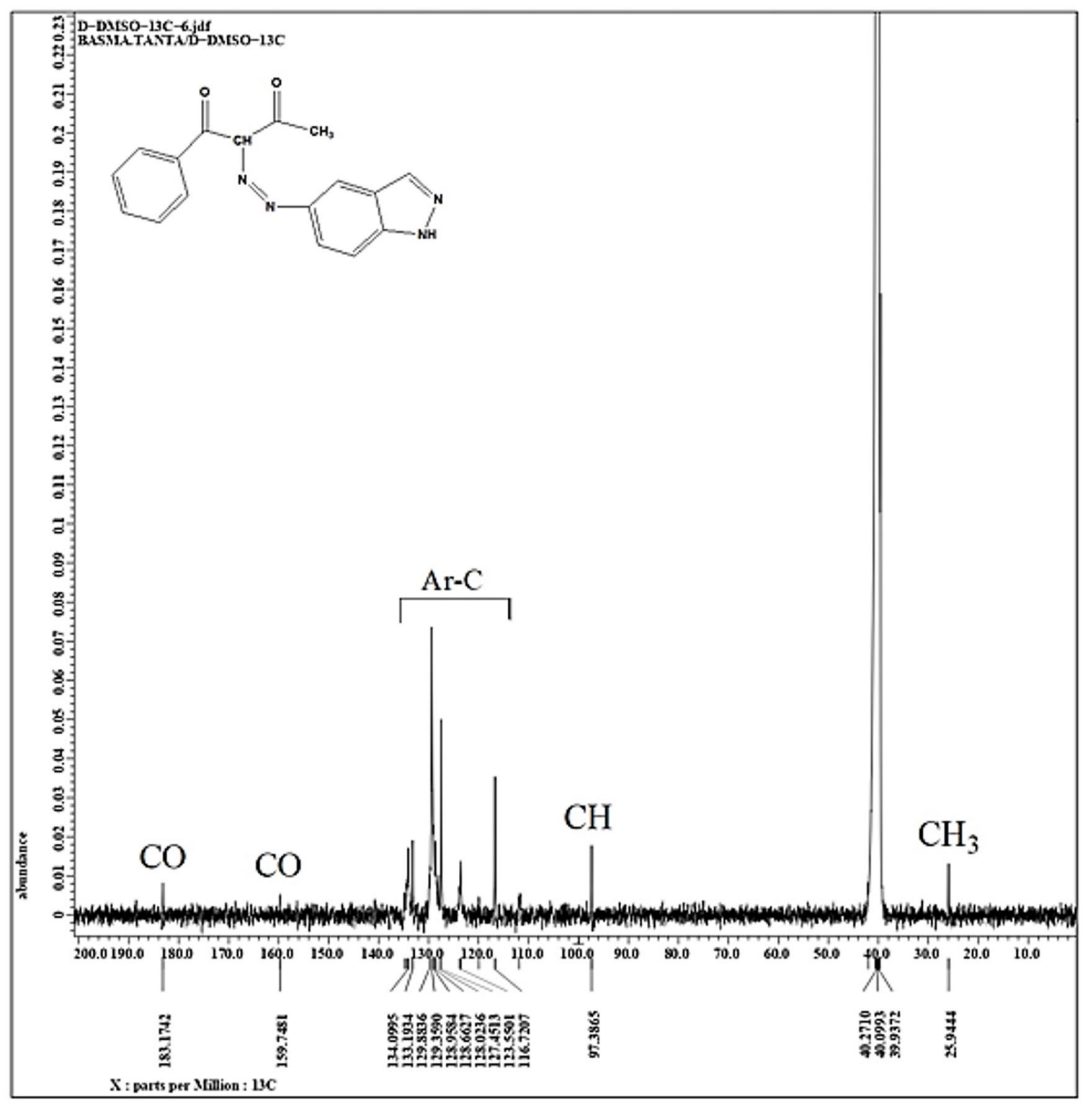
^13^C NMR spectrum of Ligand.

The ^1^H-NMR spectra of **L** were recorded in DMSO-d_6_ and exhibit a doublet signal at δ(ppm): 8.35 and two triplet signals at 7.57 and 7.51 due to the phenyl ring’s aromatic protons. The two doublet signals 7.92 and 7.80, and the singlet signals 8.01 and 7.63 belong to the indazole protons. Singlet signal at δ(ppm):6.52 that belongs to the NH proton (disappeared with deuteration). The aliphatic protons show two singlet signals at 4.26 and 2.16, which correspond to CH and CH_3_ protons, respectively (Fig. 2)^35,36^. Additionally, the ^13^C-NMR spectrum of **L** exhibited two signals at δ(ppm):183.17 and 159.74 related to 2CO groups. The signals in the range δ(ppm): 116.72-134.09 are assigned to the phenyl and indazole carbons. Further CH and CH_3_ carbon atoms appeared at δ(ppm): 97.38 and 25.94, respectively. (Fig. 3)

#### Infrared spectra

**Fig. 4:**
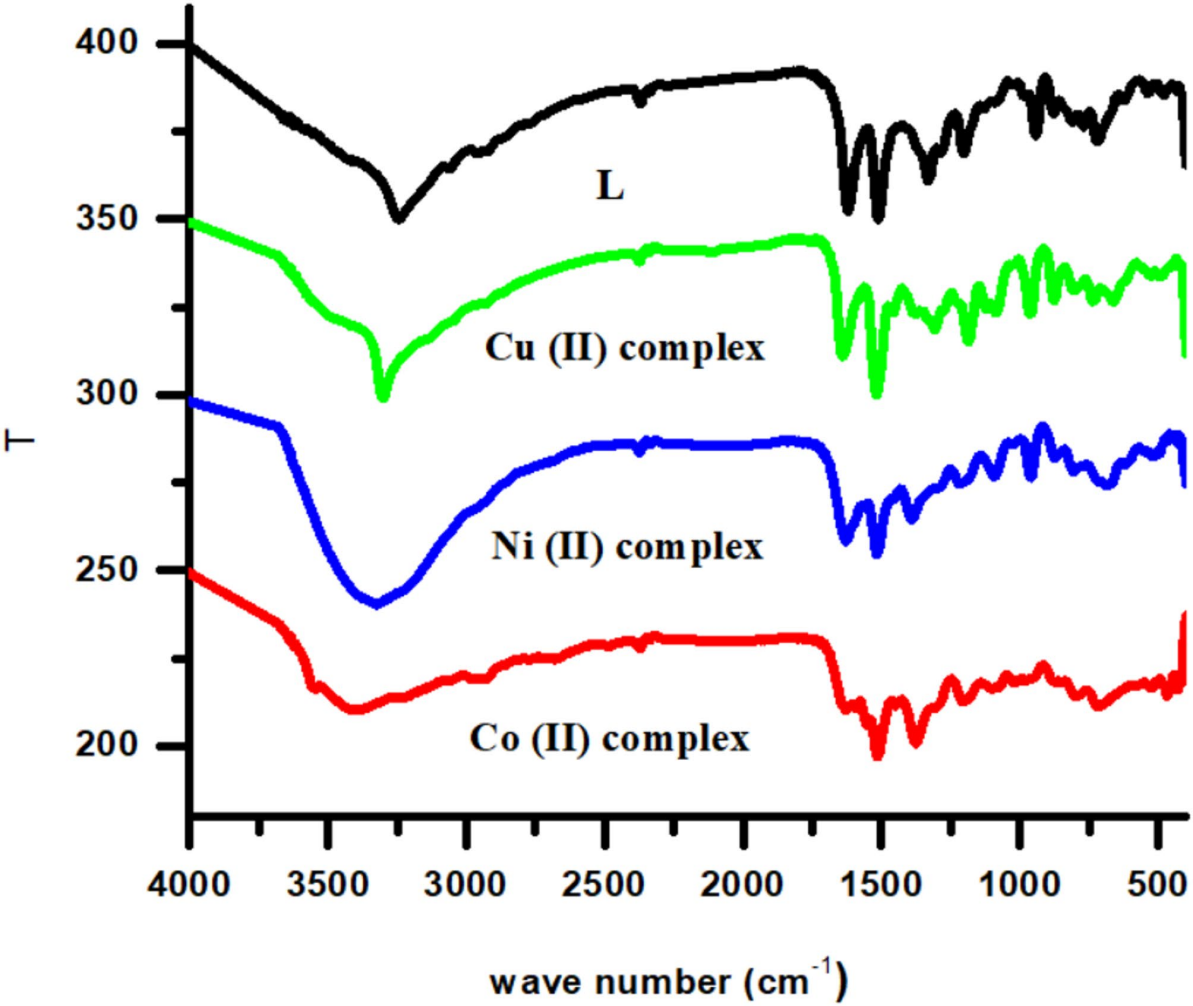
IR spectra of L and Co(II), Ni(II), and Cu(II) complexes.

The structures of the metal complexes can be easily recognized by comparing the ligands’ and their metal complexes’ infrared spectra, as shown in Table 2; Fig. 4. The IR spectrum of the free ligand (**L**) displays a stretching at 1553 cm^− 1^ that is assigned to the carbonyl group (CH_3_CO)^6^. This band exists in each complex in the same region. This means that the Oxygen atom in the acetyl group is not participating in the complex formation. Further, the bands of NH and C = N of the indazole moiety appeared at 2926 and 1510 cm^− 1^, respectively. These bands showed a slight shift in the spectra of all complexes, 1–7 cm^− 1^. This confirms that these groups are not involved in complexation. However, the free ligand displays a band at 1619 cm^− 1^ that is devoted to the carbonyl of the benzoyl group. It is shifted to lower or higher wavenumbers by 12–23 cm^− 1^, suggesting the participation of this group in the chelation process. The band at 1415 cm^− 1^ is due to the azo group of **L**. This band is shifted to higher wavenumbers, 34–41 cm^− 1^, upon complex formation because of the nitrogen azo group’s cooperation with the metal center. These assignments are supported by the development of new bands at 585– 542 and 451–444 cm^− 1,^ which correspond to the M-O and M-N, respectively. The broadband within the 3300–3318 cm^− 1^ range, which is assigned to the OH group of water molecules.

**Table 2:** IR spectral data (cm^− 1^) of the ligand and Co (II), Ni (II) and Cu (II) complexes.

Compound	ν _OH_	ν _NH_	ν _C=O_ (Benzene ring)	ν _C=O_ (Methyl group)	ν _C=*N*_	ν _*N*=*N*_	ν _M−O_	ν _M−*N*_
L	–	2926	1619	1553	1510	1415	–	–
Co (II) complex	3318	2925	1635	1552	1513	1449	577	450
Ni (II) complex	3322	2923	1631	1553	1515	1456 *(sh)*	543	453
Cu (II) complex	3300	2925	1642	1552 *(b)*	1517	1455	553	451

*sh*: shoulder.

*b*: broad.

#### Thermal gravimetric analysis (TGA)

**Fig. 5:**
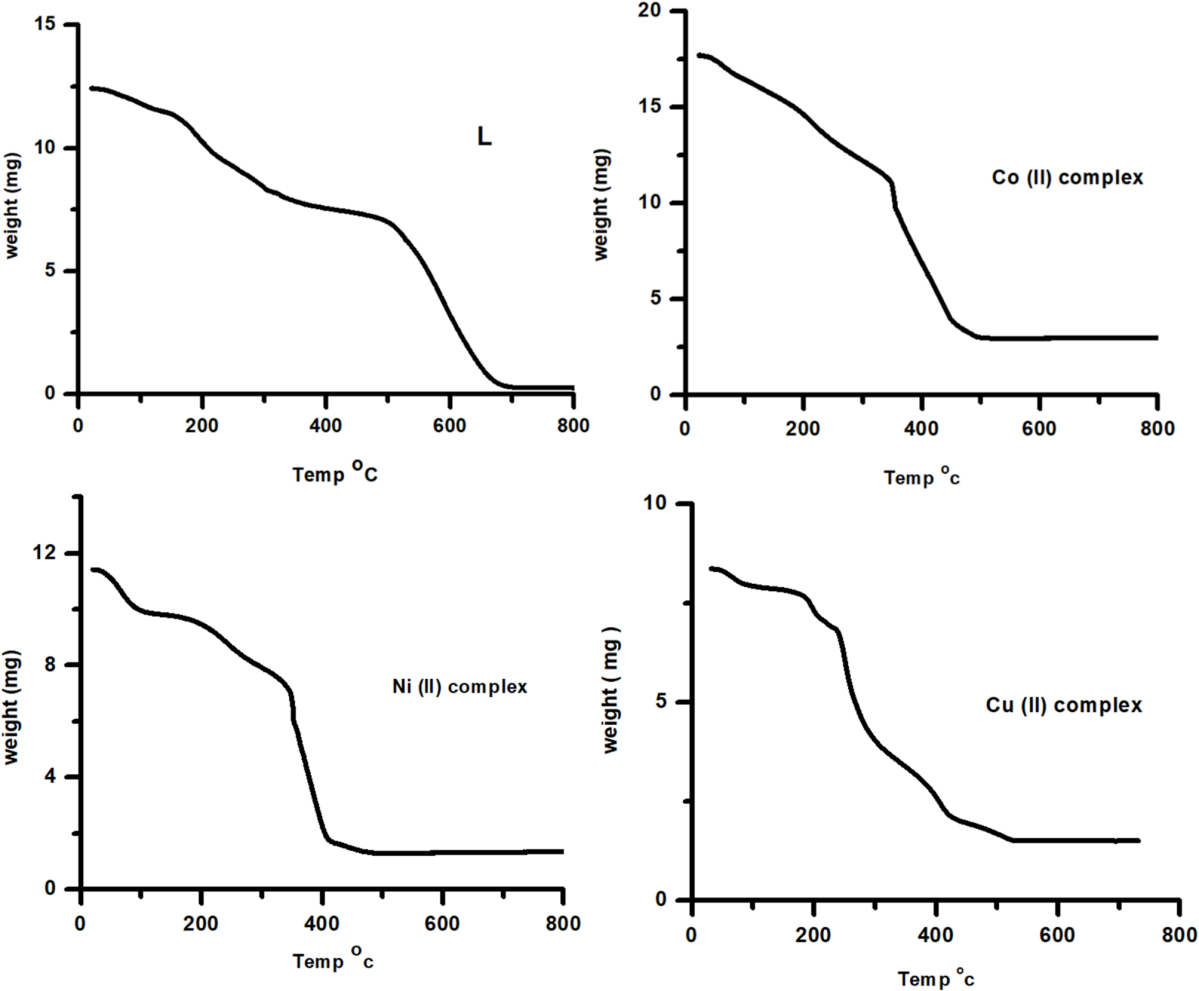
TGA thermogram of L and its metal complexes.

**Fig. 6:**
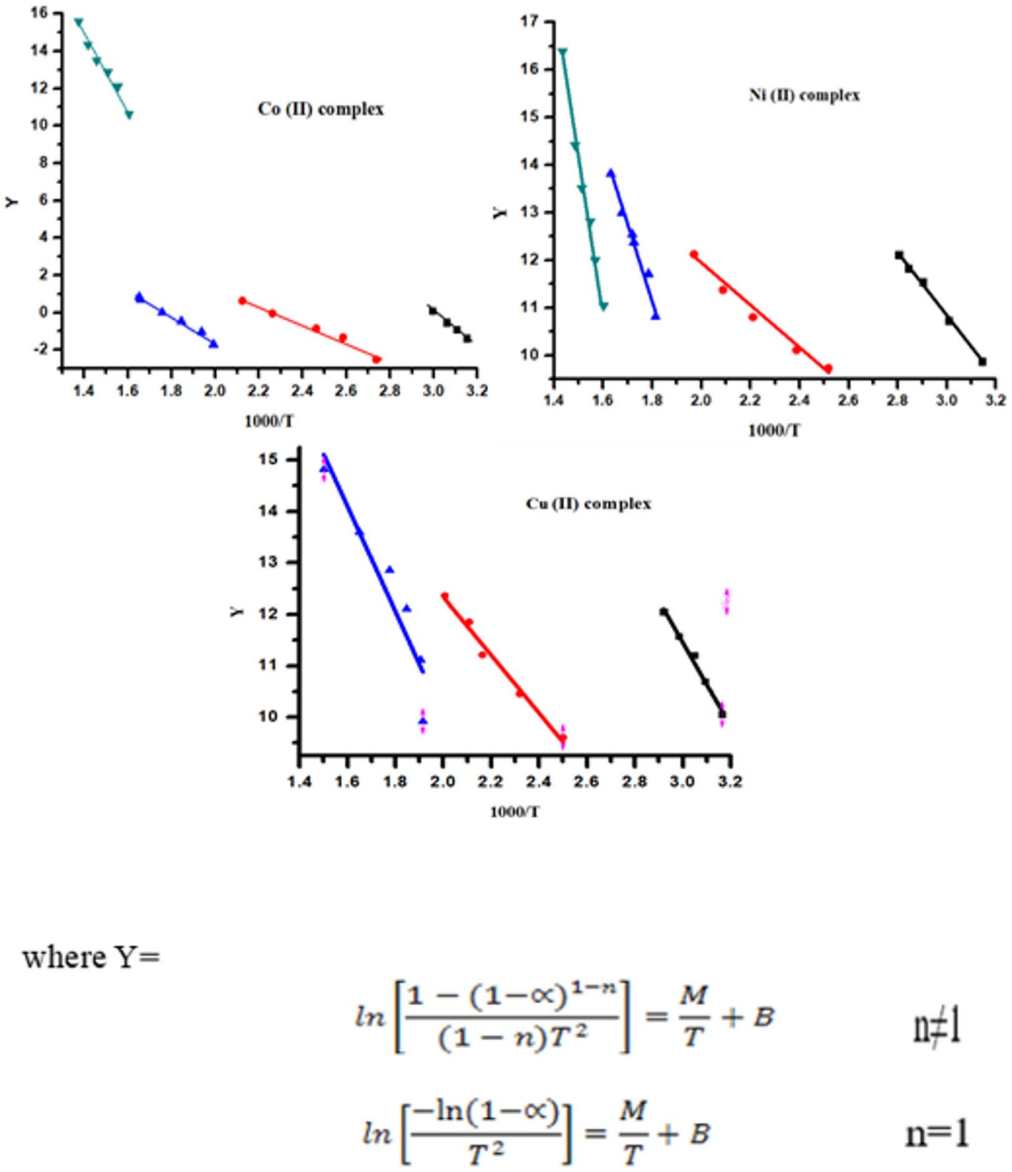
Coats-Redfern plots for the decomposition of metal chelates, where the 1st stage is black, the 2nd stage is red, the 3rd is blue, and the 4th is green

Thermogravimetric analysis was performed on the prepared complexes to determine their thermal stabilities and to confirm or deny the presence of solvent in their structures^37^. Measurements were performed at heating rates of 10 °C min^− 1^ under a nitrogen atmosphere. The thermo-analytical data of metal complexes are given in Table 3. The thermograms are shown in Fig. 5. As seen clearly from Table 3. The Co(II) and Ni(II) complexes went through four stages of thermal breakdown, while the Cu(II) complex has three phases of degradation that adopt a different coordination geometry. The TGA curve of azo-dye confirms thermal stability up to 120 °C, consistent with its melting point. For all three complexes, the first decomposition stage (27–81 °C) corresponds to loss of lattice H_2_O molecules. These relatively low temperature of this stage confirms that these water molecules are weakly held outside the coordination sphere. The second stage in Co(II) and Ni(II) (79–154 °C and 74–178 °C, respectively) is attributed to coordinated water loss. These higher temperatures reflect the stronger metal-oxygen bond formed between the water molecule and the metal center. The chloride ion loss observed in the third stage for all complexes (154–289 °C) at relatively high temperatures indicates that the chloride ions are directly coordinated to the metal center. The final decomposition stage (259–520 °C) in all complexes corresponds to the breakdown of organic ligands, yielding thermally stable metal oxide (CoO, NiO, CuO) as final residues. The high temperatures required for this stage reflect the thermal robustness of the aromatic azo-dye ligand framework, which requires significant energy to fully decompose. The Coats-Redfern methodology^38^ was used to determine the reaction’s thermodynamic parameters and the order, as seen in Fig. 6; Table 4. The complexes under study are very stable, as indicated by the high activation energy (E*). Gibbs free energy (G*) values, which are positive, suggest that the reactions were nonspontaneous. However, the endothermic nature of the pathways of degradation is confirmed by the positive enthalpy (H*). Additionally, smaller or negative entropy (S*) values suggest a more ordered complex than the reactant or extremely slow breakdown reactions^39^.

**Table 3:** Thermogravimetric analysis of ligand and its metal chelates.

Compound	Temp °C	Wt. loss%		Assignment
		Calc.	Found	
Co(II) complex	27–79	5.40	5.29	Loss of 1.5 hydrated water
	79–154	7.21	7.27	Loss of 2 coordinated water
	154–259	14.22	14.14	Loss of 2 Chloride ions
	259–490	58.19	58.09	Complete decomposition of the organic part
		14.98	15.21	Formation of CoO as the final product
Ni(II) complex	27–74	8.70	8.93	Loss of 2.5 hydrated water
	74–178	6.96	6.92	Loss of 2 coordinated water
	178–289	13.73	13.67	Loss of 2 chloride ions
	289–480	56.31	56.9	Complete decomposition of the organic part
		14.3	13.58	Formation of NiO as a final product
Cu(II) complex	27–81	3.92	4.05	Loss of 1 hydrated water
	81–151	–	–	Thermal stability
	151–242	15.47	15.26	Loss of 2 chloride ions
	242–520	63.31	62.89	Complete decomposition of the organic part
		17.32	17.8	Formation of CuO as the final product

**Table 4:** Decomposition steps and activation parameters of the metal chelates.

Compound	Step	*n*	*R*	E^*a^	ΔH^*a^	(− ΔS^*b^)	ΔG^*a^	Ts
Co(II) complex	1st	1	-0.9986	81.940	79.261	0.0965	110.365	322.22
	2nd	1	-0.9899	40.633	37.189	-0.0659	9.908	414.26
	3rd	1	-0.9942	57.614	52.990	-0.0556	22.070	556.167
Ni(II) complex	1st	0.66	-0.9982	55.6173	52.7846	0.1031	87.927	340.714
	2nd	0	-0.9911	35.8593	32.109	0.0105	36.862	450.992
	3rd	2	-0.9884	126.774	121.948	0.1704	220.833	580.397
Cu(II) complex	1st	2	-0.9922	71.312	68.575	0.1582	120.646	329.096
	2nd	0	-0.9919	46.825	43.056	0.0387	60.623	453.28
	3rd	2	-0.946	84.878	80.133	0.0985	136.397	570.677

^*a^(KJ.mol^− 1^.K^− 1^).

^*b^(KJ.mol).

#### Electronic absorption spectra and magnetic susceptibility

**Fig. 7:**
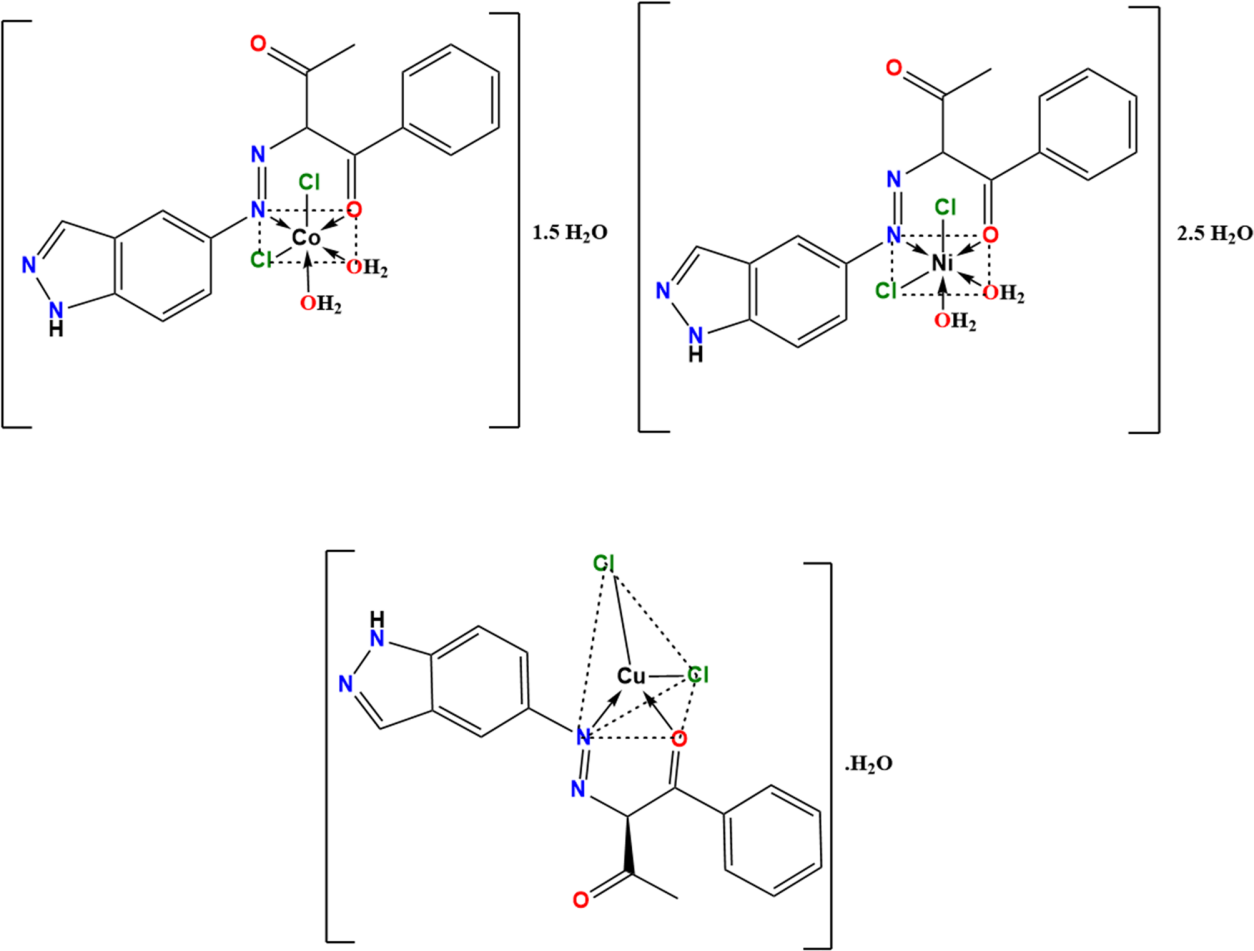
The structures of the synthesized Co(II), Ni(II), and Cu(II) complexes.

The electronic absorption spectra of complexes were studied in Nujol mull. The spectrum of Co(II) complex displays two bands at 188,679 and 142,857 cm^− 1^ due to the ^4^T_1_g(F) → ^4^A_2_g and ^4^T_1_g(F)→ ^4^T_1_g(p) transitions, respectively. This suggests an octahedral arrangement surrounding the Co(II) ion^40,41^. The Ni(II) complex shows two bands at 19,607 and 142,653 cm^− 1,^ which are attributed to the ^3^A_2_ →^3^T_1_ (F) and ^3^A_2_ →^3^T_1_(P) transitions, respectively. This indicates that the Ni(II) ion is surrounded by an octahedral configuration. The spectral band at 14,388 cm^− 1^, considering tetrahedral geometry around Cu(II) ion, is recognized as the ^2^T_2_→ ^2^E transition in Cu complex spectrum. The magnetic moment data acquired at room temperature indicate that all of the solid complexes are paramagnetic. The values are 4.05, 3.11, and 1.82 BM for Co(II), Ni(II), and Cu(II) complexes. These values are greater than the theoretical spin value regarding the orbital contribution^42^. Metal complexes structures are shown in Fig. 7, which are based on the elemental analysis data, ICP, TGA, FT-IR, UV-Vis, magnetic moment, and conductance results.

#### XRD analysis

**Fig. 8:**
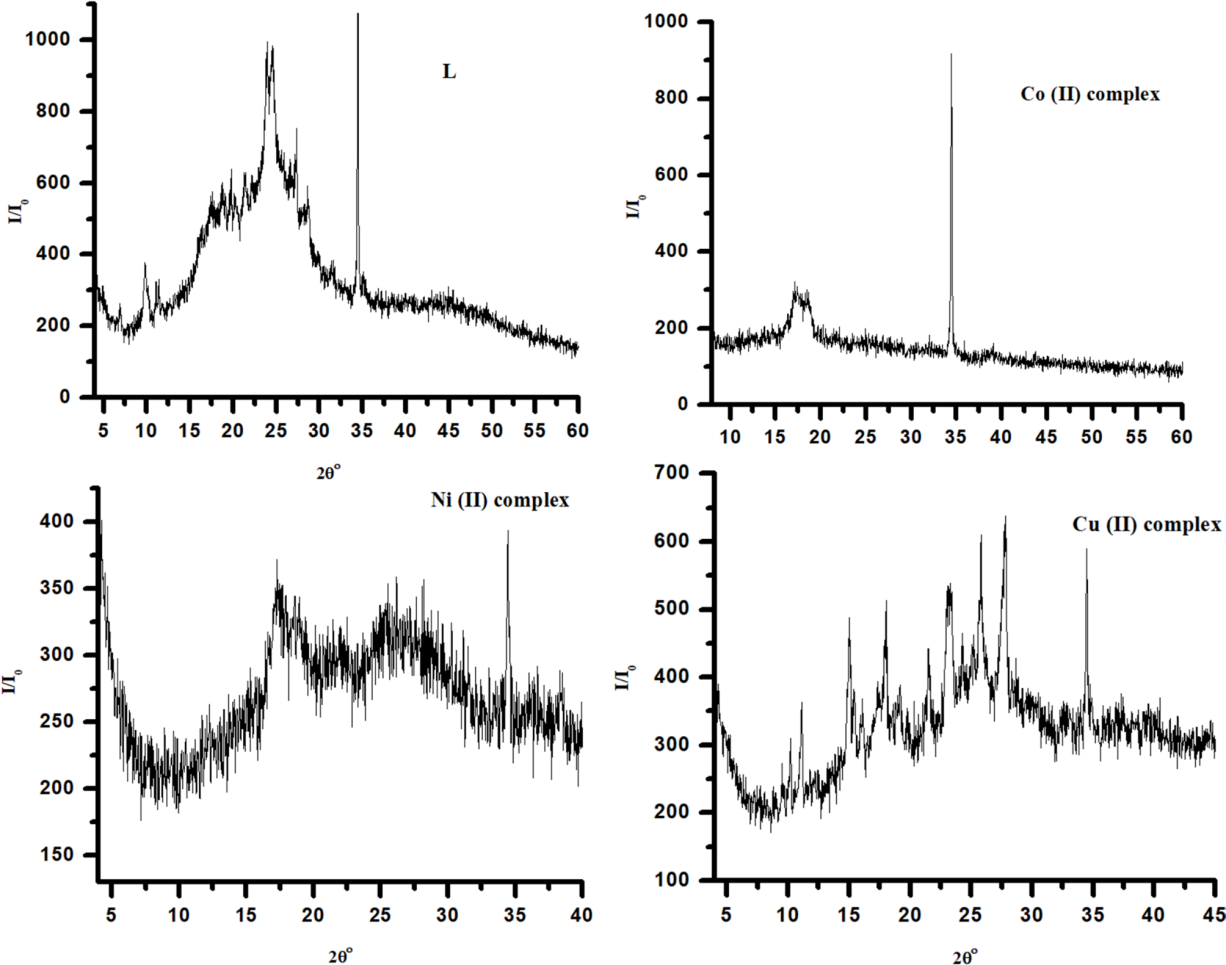
XRD diffraction patterns of L and its complexes.

The azo-dye ligand and its divalent cobalt, nickel, and copper complexes XRD patterns were measured within the range 2θ = 3º-90º. The ligand and metal complexes’ X-ray diffractograms are displayed in Fig. 8. The powder XRD patterns of the complexes and the starting ligand show an entirely different variance in the data. This outcome implies that new complexes were formed by the coordination of the metal ions with the ligand. The patterns of XRD indicate that the crystals of the complexes are not ideal, but they possess both crystalline and amorphous characteristics^43^. Also, XRD showed that the nature of the azo-dye ligand is semi-crystalline, while the Co(II) complex is crystalline. The XRD phase behavior of the Ni(II) complex is wide-bumps with lower intensities, and scattered in all directions, which proves an amorphous phase for this complex. The Cu(II) complex has several distinct peaks, confirming that this complex has a polycrystalline phase^44,45^. To find the average particle size of the crystalline metal complexes, Scherrer’s formula was applied:

$$\:D=\frac{K\lambda\:}{{cos}\theta\:}$$

Where (λ) is the wavelength of x-ray radiation (0.15406 nm), (θ) is the Bragg’s diffraction angle, (K) is the Scherrer constant, and (β) is the FWHM of the peak (full width at half maximum of the reflection peak with the identical highest intensity in the diffraction pattern). For spherically crystalline particles, the Scherrer constant (K) is usually taken to be 0.9. The average particle size of the complexes is given in Table 5.

**Table 5:** XRD data of the azo-dye and its metal complexes.

Compounds	2theta (^o^)	d(A^o^)	I/I_o_	FWHM (^o^)	D (nm)	Average (nm)
Azo-dye	24.54	3.6249	1000	0.360	22.583	17.75
	23.93	3.7157	973.66	0.360	22.557	
	16.39	5.4028	760.96	0.9889	8.116	
Co(II) complex	34.48	2.5992	1060	0.1877	44.315	24.7
	17.18	5.1576	625.68	0.540	14.878	
	18.52	4.7877	492.90	0.540	14.905	
Cu(II) complex	26.65	3.3428	1820.45	1.080	7.559	8.44
	23.24	3.8237	1640.65	0.785	10.331	
	17.39	5.0956	1008.20	1.080	7.441	

### Primary study of atmospheric corrosion

#### Weight loss method

The weight loss measurements were made to calculate the atmospheric corrosion rates of untreated and treated steel specimens by using the following formula:

$$\:{\mathrm{Corrosion}}\:{\mathrm{rate}}\left( R \right) = \frac{{W_{0} - W_{t} }}{{At}}$$

In which W_0_ and W_t_ represent the weight of the specimens before and after exposure to the atmosphere (g), A is the surface area (cm^2^) of each specimen, and t is the exposure time. The following formula was used to get the percentage inhibition efficiency (%IE):

$$\:\%\:IE=[1-\frac{R\:treat}{R\:untreat\:}]\times\:100$$

The depth of the corrosive surface of the specimen (L) in µm is determined from the following equation:

$$\:L=\frac{\varDelta\:w}{Ad}$$

**Fig. 9:**
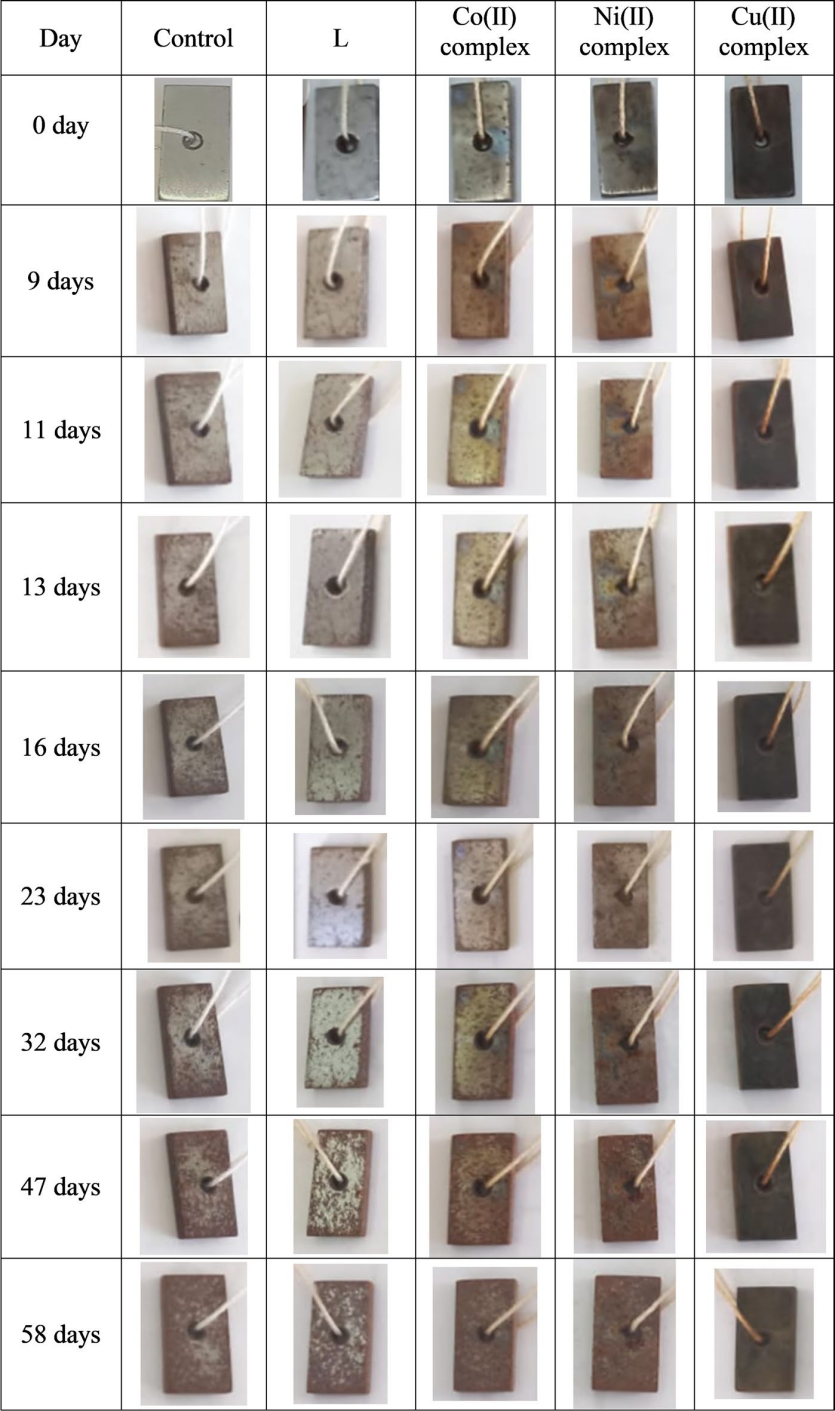
Photograph of treated and untreated steel specimens after 58 days of atmospheric exposure.

Where d is the standard density of steel (7.87 g/cm^3^). The rates of corrosion of treated and untreated specimens are denoted R_treat_ and R_untreat_. The values of R and % IE from the weight loss calculations after 58 days are summarized in Table 6. The R values decrease after using the inhibitor from 0.0519 to 0.0121 − 0.0028 g/cm^2^.day for untreated and treated specimens, respectively. This indicates the inhibiting effect of these compounds. Additionally, the data display the depth of the corrosive layer in the range of 9.2855–2.1992 × 10^− 5^ μm. After 58 days of atmospheric exposure, the results of the untreated and treated specimens are shown in Fig. 9. We noticed that after removing the samples from the inhibitor solutions, there was a slight change in the color of the specimens, except for the Cu(II) complex. Where the color change was more obvious. A discoloration on the steel samples and a more heterogeneous surface appearance and coarser granulometry can be seen with time. While the specimen treated with Cu(II) complex did not exhibit such discoloration. This confirms the previous results obtained through the weight loss study. The superior performance of the Cu(II) complex can be explained by its unique electronic configurations, which facilitate stronger coordination interaction with the steel surface through back-donation of electrons.

**Table 6:** The weight loss, corrosion rate, inhibition efficiency, and depth of corrosion layer of iron of the synthesized compounds.

Compounds	Weight loss (g)	Corrosion rate (g/cm^2^.day)	IE%	L × 10^−5^ (µm)
Control	0.0162	0.0519	----	39.5856
Ligand	0.0037	0.0118	77.26	9.0423
Co(II) complex	0.0038	0.0121	76.68	9.2855
Ni(II) complex	0.0036	0.0115	77.84	8.79679
Cu(II) complex	0.0009	0.0028	94.60	2.1992

#### Molecular Dynamics (MD) simulations

**Fig. 10:**
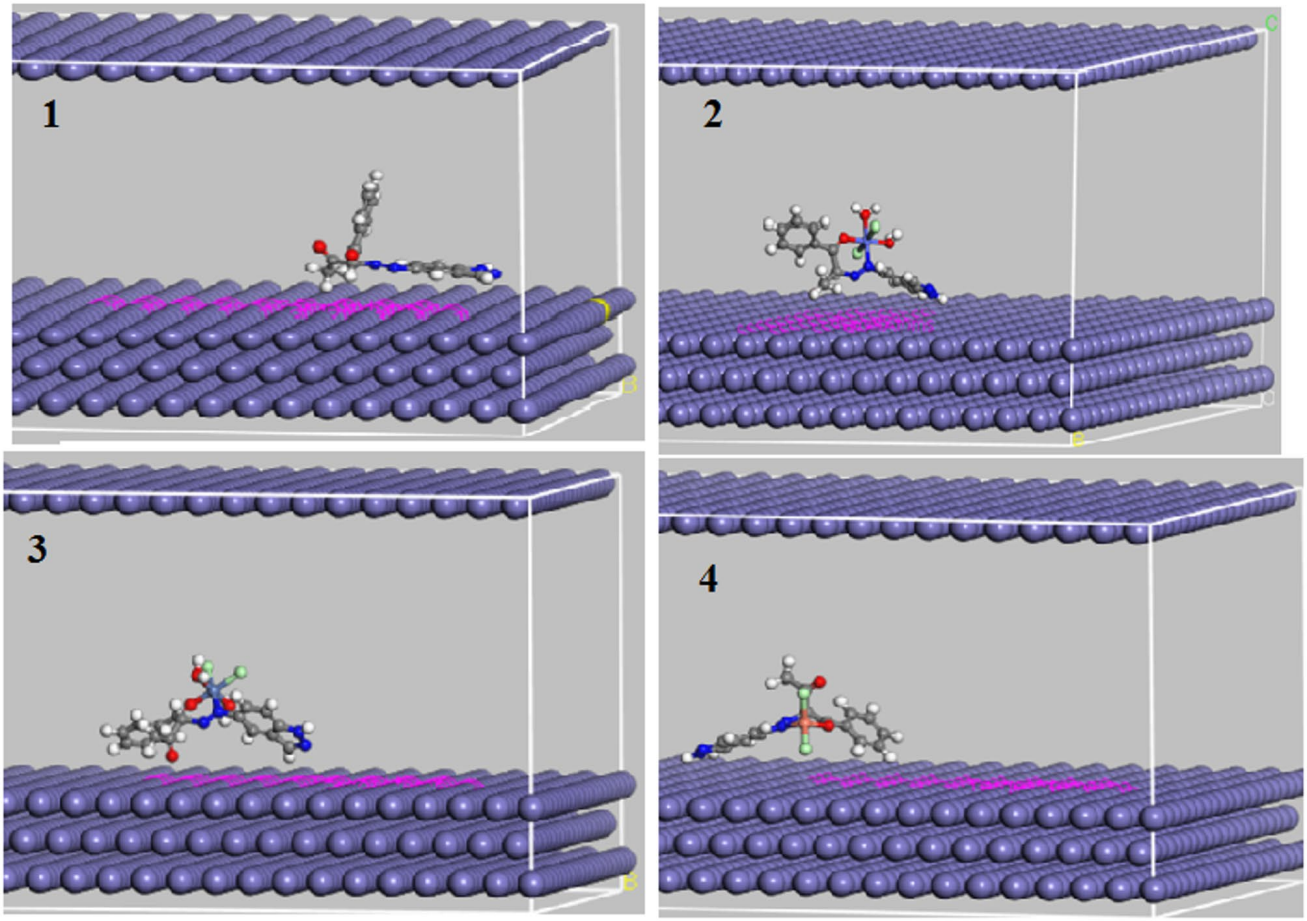
MD models of compounds on the Fe(110) surface showing (1) L, (2) Co(II) complex, (3) Ni(II) complex, and (4) Cu(II) complex.

To understand the adsorption mechanism between the inhibitor structure and its inhibition behavior on the Fe(110) surface, the quantum chemical calculation using the forcite quench dynamics module and adsorption locator of the Material Studio Program has been utilized^46^. The resulting adsorption energy, rigid absorption, and deformation energies are summarized in Table 7. The adsorption energy serves as a key descriptor for evaluating the strength of inhibitor surface interactions. The negative adsorption energy values obtained for all the inhibitors suggest a favorable interaction between the inhibitor molecules and the metal surface. In this context, the deformation energy refers to energy released upon relaxation of the adsorbed inhibitor-adsorbate system on the metal surface. The rigid adsorption energy represents the energy liberated when inhibitor molecules adsorb onto the metal surface without geometric relaxation^32^. As reported in Table 7, the adsorption energy was computed in the range of (-3.621, -4.074) kcal/mol. The geometrically optimized configurations of the synthesized compounds adsorbed on the Fe(110) surface are illustrated in Fig. 10. All complex molecules adopted a parallel orientation relative to the metal surface. In the case of the free ligand, the indazole ring, carbonyl group, and methyl group maintained a planar orientation, where the aromatic ring oriented perpendicularly. This planar orientation maximizes the molecular contact area with the Fe(110) surface, which is directly correlated with enhanced inhibition efficiency. From Fig. 10, the planar distances on the Fe(110) surface are 10.226, 9.877, 13.063, and 14.084 °A. The vertical distances between the inhibitor molecules and the Fe(110) surface are 4.162, 6.161, 3.450, and 2.450 °A, for the L, Co(II), Ni(II), and Cu(II) complexes, respectively. A greater planar distance combined with a shorter vertical separation corresponds to higher inhibition efficiency. The primary adsorption sites for all complexes were identified as the indazole ring and the azo group. Additional contributions from the aromatic ring, the carbonyl group, and the chloride atom were observed for the Cu(II) and the Ni(II) complex, respectively. Based on the data presented in Tables 6 and 7, and Fig. 10, the inhibition activity follows the order: Cu(II) > Ni(II) > ligand > Co(II), a trend consistent with the weight-loss measurements. The adsorption of the present inhibitors on the Fe(110) surface is classified as physisorption, as evidenced by several key indicators. The negative adsorption energy values ranging from (-3.62 to -4.07 kcal/mol), indicate a tendency for physisorption dominated by non-covalent interactions such as van der Waals forces^47^. All molecules adopted a flat-lying (parallel) orientation relative to the metal surface, maximizing the molecular contact area and enhancing the inhibition efficiency^48^. The vertical distance (2.450–6.161 °A) between the inhibitor molecules and the Fe(110) surface falls within the characteristic physisorption. The negligible small deformation energy values (0.007–0.077 kcal/mol), confirm that no structural distortion occurs upon adsorption.

**Table 7:** List of molecular dynamics simulation parameters (kcal/mol) of compounds on the Fe(110) surface.

Compounds	Adsorption energy	Rigid adsorption energy	Deformation energy
L	− 3.71533666	− 3.79293894	0.07760229
Co(II) complex	− 3.62167027	− 3.65197885	0.03030858
Ni(II) complex	− 3.74395615	− 3.75193442	0.00797827
Cu(II) complex	− 4.07460329	− 4.12193779	0.04733450

### SEM and EDX

**Fig. 11:**
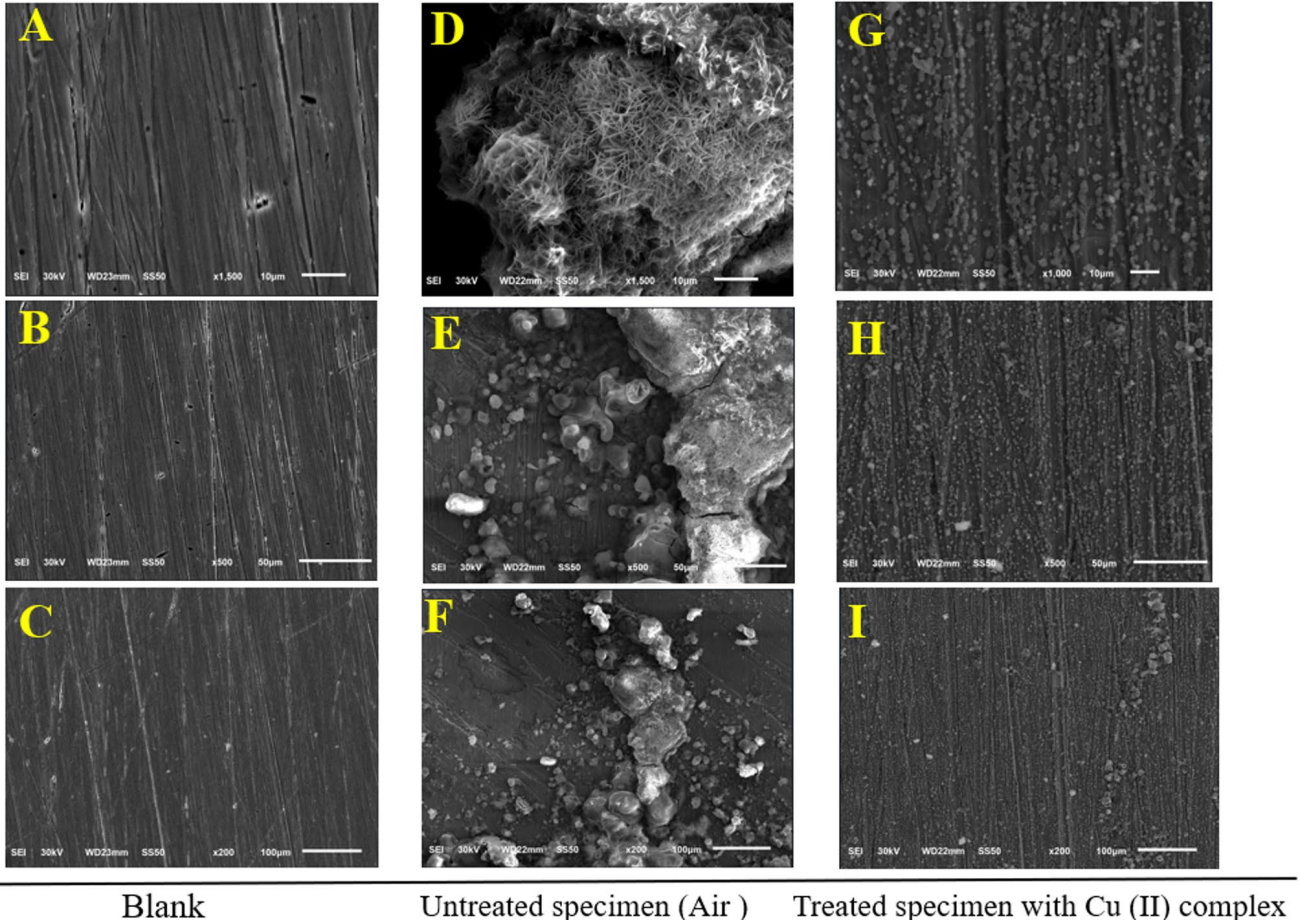
Micrographs of iron specimens.

**Fig. 12:**
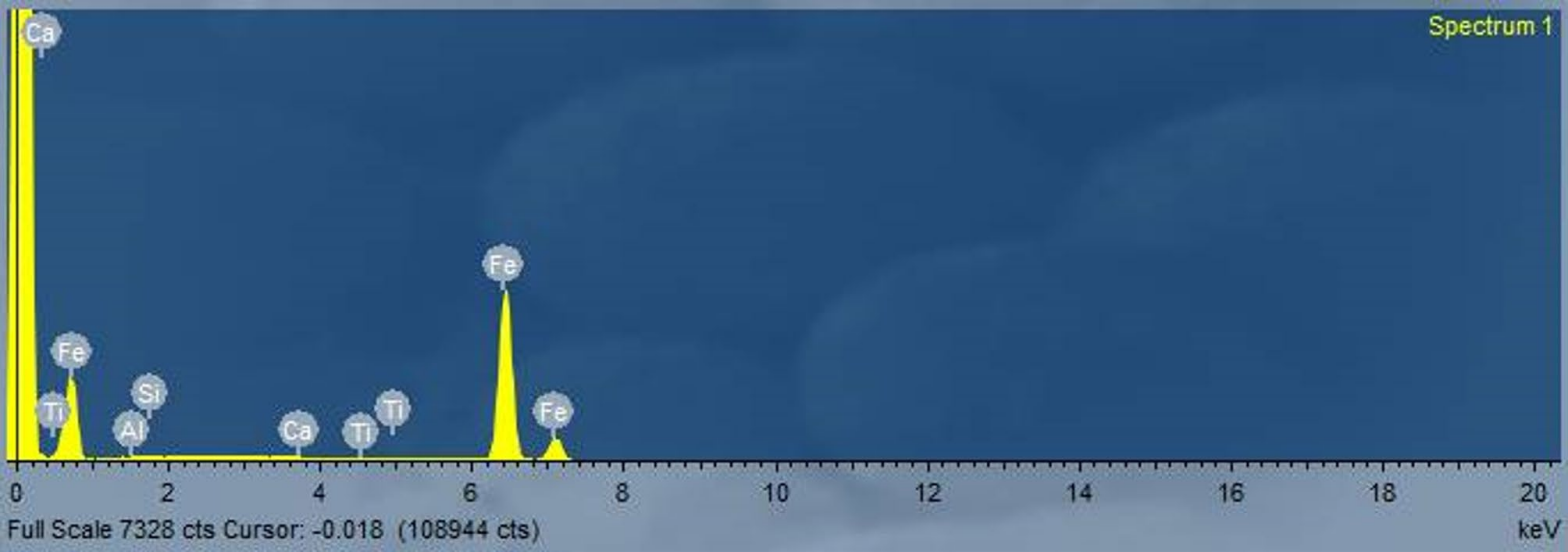
EDX spectrum of pure iron specimen.

Figure 11 displays the images of SEM micrographs of the blank iron specimen (A - C), in the absence of inhibitor (D - F), and in the presence of Cu(II) complex as inhibitor at the ideal concentration of (10^− 3^ M) from (G - I). The SEM results showed that the iron surface exposed to the atmosphere without an inhibitor has rough pits, cracks, and casual scratches, i.e., the surface becomes damaged and corroded. However, in the presence of the inhibitor Cu(II) complex, the surface of iron is relatively smoothed, due to the formation of a protective film on the iron surface. EDX for a pure iron specimen is given in Fig. 12, which shows the main elements of the iron specimen, which are Fe (99.32%), Si (0.36%), Ti (0.18%), Al (0.08%), and Ca (0.07%).

### Computational study

#### Energy optimization by DFT theory

**Fig. 13:**
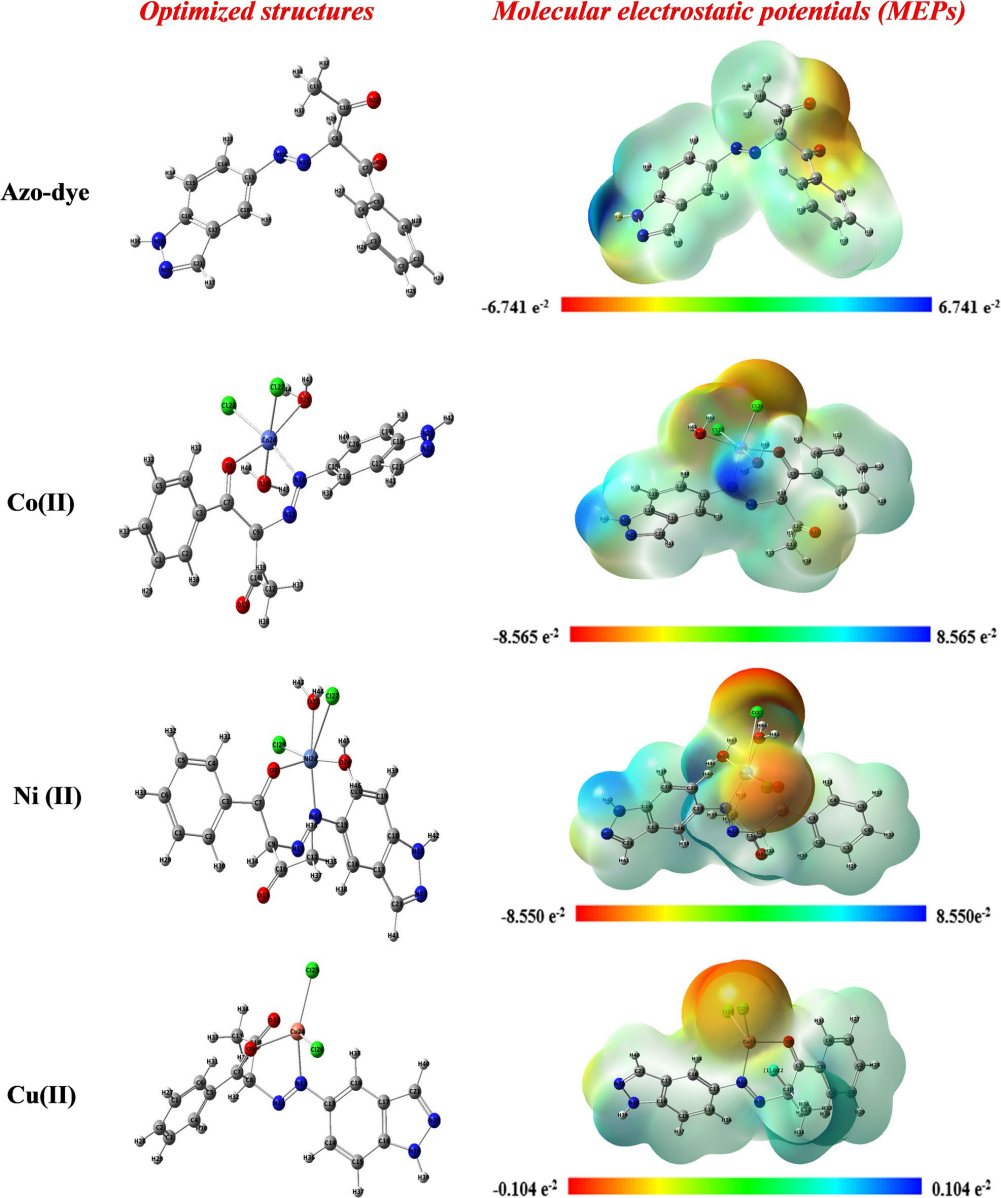
The optimized molecular structures and molecular electrostatic potentials (MEPs) of the ligand and its metal chelates.

The electronic composition of the compounds can be accurately and successfully predicted using the computational study. The DFT gives essential information regarding a molecule’s structure, stability, and reactivity^27–29^. The optimized molecular structures are displayed in Fig. 13, which confirms that the ligand’s O9 and N12 atoms can chelate with the metal ion. In the gaseous state, the DFT technique fully optimizes the geometries of the ligand’s singlet ground state and its divalent cobalt, nickel, and copper complexes. The following observations have been achieved as a result of the optimized molecular geometry:

1. All of the active coordination bonds had various dimensions than the free ligand moiety. This implies that there is novel bonds like M-N and M-O are formed. Also, elongation of the C-N bond and shortening of the C-O bond due to the chelation process.
2. Co(II) and Ni(II) complexes are octahedral, according to the calculated bond angles of the coordination sphere surrounding the center of the Co(II) and Ni(II) ions, which fall between 78.20 and 91.81 for Co(II), and from 47.11 to 93.67 in Ni(II) for the right angle. The linear angle is within the range of 167.5–174.9 for Co(II) and 164.88–168.30 for Ni(II). These bond angle values approach the standard octahedral values for Co(II), but there is some distortion in the Ni(II) complex. The experimental results and all theoretical data correlate perfectly.
3. The coordination sphere bond angles around the Cu(II) metal ion center range from 85.93 to 120.12, indicating that the Cu(II) complex is tetrahedral. Because they differ from the typical tetrahedral angles, the complex geometry is distorted.

#### Mulliken charge population analysis

**Fig. 14:**
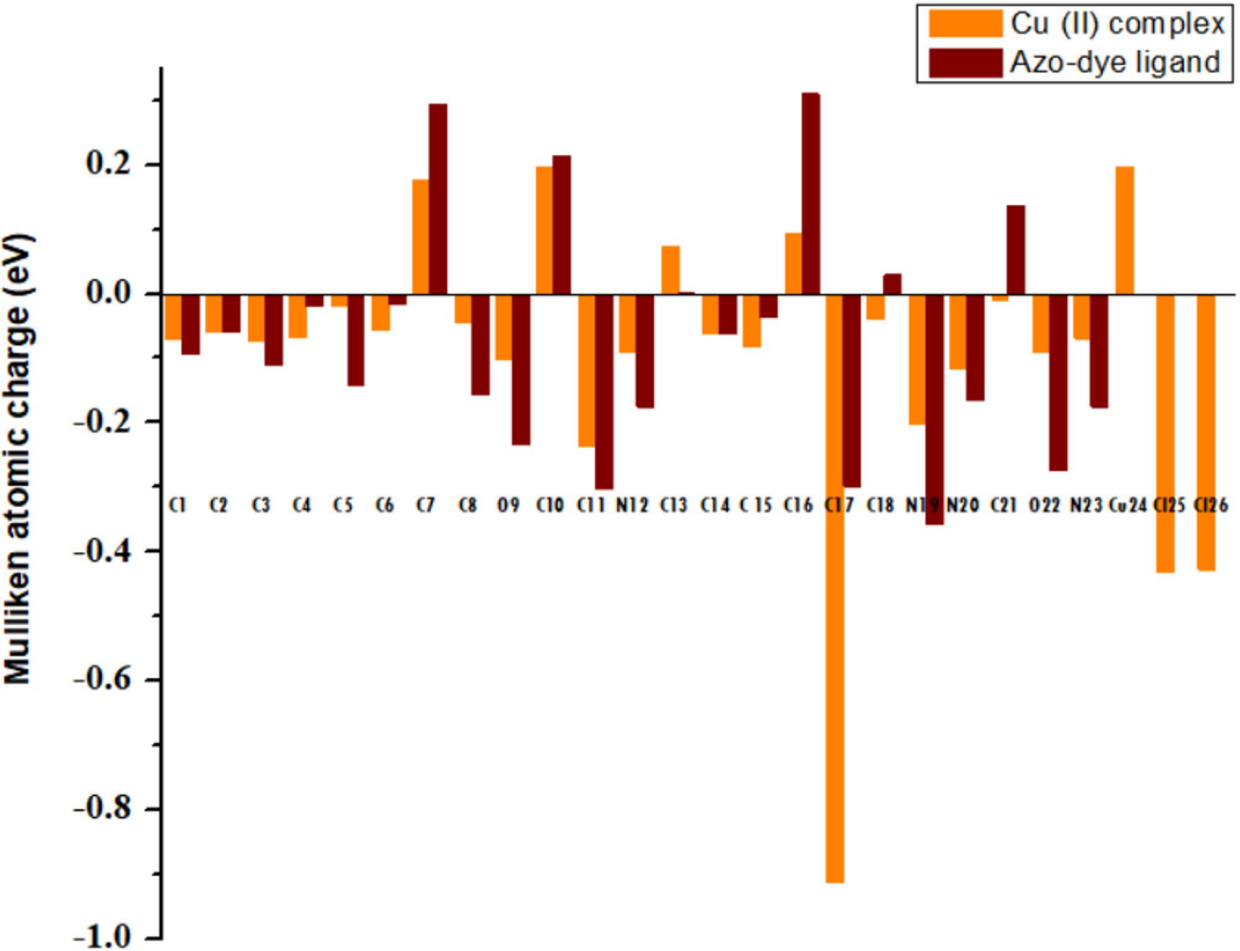
Mulliken atomic charge distribution of the ligand (B3LYP/6-311^++^G (d, p) and Cu(II) complex (B3LYP /LanL2MB) basis set.

Mulliken charge is an essential factor in molecular structure determination. Positive charges are present in most carbon atoms and hydrogen atoms, which enable them to accept electrons. However, nitrogen, oxygen, and carbon atoms attached to hydrogen have a negative charge, and thus, they are able to provide electrons. The O9 and N12 atoms have a greater electrophilic charge density in the free ligand than the Cu(II) chelate, as displayed in Fig. 14. As compared to the ligand, the atomic charge clearly changes during chelation, as shown in Table 8. Atomic Mulliken charges, bond lengths, and bond angles are all listed in Tables S1- S5.

**Table 8:** The obvious change in the atomic charge upon chelation.

Ligand		Co(II) complex		Ni(II) complex		Cu(II) complex	
Atom	Charge	Atom	Charge	Atom	Charge	Atom	Charge
C7	0.294	C7	0.251	C7	0.201	C7	0.177
C8	− 0.156	C9	− 0.297	C9	− 0.034	C8	− 0.043
O9	− 0.234	O8	− 0.244	O8	− 0.103	O9	− 0.103
C11	− 0.303	C12	− 0.706	C12	− 0.241	C11	− 0.235
N12	− 0.175	N14	− 0.198	N14	− 0.091	N12	− 0.090
C13	0.001	C15	0.429	C15	0.071	C13	0.074
C15	− 0.037	C19	− 0.349	C19	− 0.078	C15	− 0.082
C16	0.310	C18	0.271	C18	0.092	C16	0.093
C17	− 0.298	C17	0.239	C17	− 0.019	C17	− 0.913
C18	0.029	C16	− 0.439	C16	− 0.063	C18	− 0.039
N19	− 0.358	N23	− 0.423	N23	− 0.201	N19	− 0.201
N20	− 0.164	N22	− 0.005	N22	− 0.116	N20	− 0.116
C21	0.137	C21	− 0.435	C21	− 0.018	C21	− 0.009
O22	− 0.274	O11	− 0.237	O11	− 0.140	O22	− 0.091
N23	− 0.175	N13	− 0.072	N13	− 0.061	N23	− 0.069

#### Stability and chemical reactivity

**Fig. 15:**
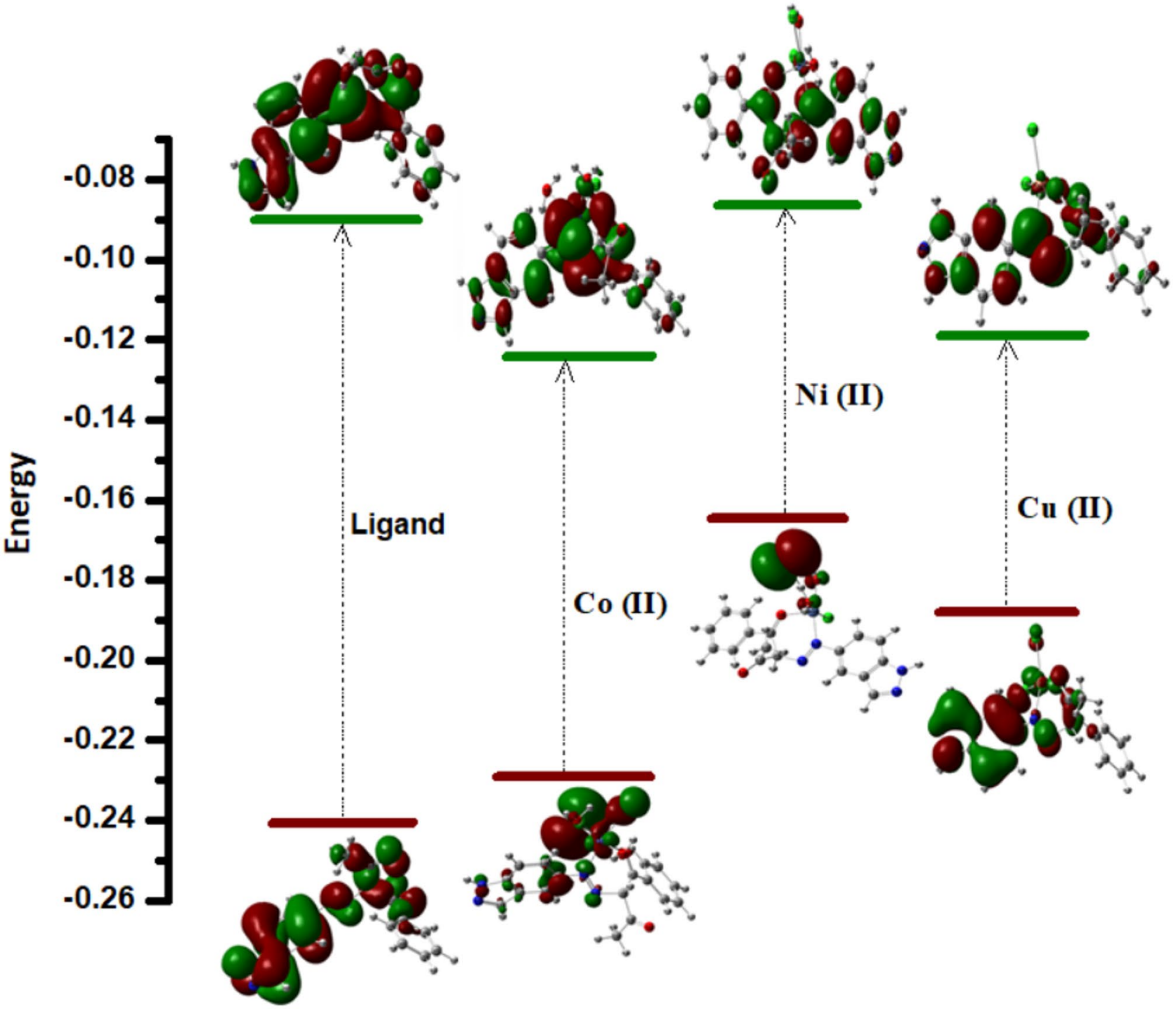
The HOMO, LUMO, and the energy gaps of the ligand and metal chelates.

The most effective method to forecast a compound’s chemical reactivity was to use the frontier molecular orbitals (**FMOs**) in combination with common descriptors^27,29^. The highest occupied molecular orbital (**E**_**HOMO**_) and lowest unoccupied molecular orbital (**E**_**LUMO**_) of inhibitor molecules react chemically with metal surfaces. Two processes dictate how the inhibitor molecules interact with the metal surface: (i) electron donation to the metal’s vacant d-orbital from the inhibitor molecule, and (ii) a back donation of electrons to the inhibitor molecule from the metal’s filled d-orbital. The two processes complement one another, and both help to improve the inhibitor molecule’s adsorption onto the metal surface. The **E**_**HOMO**_ is a measure of the inhibitor molecule’s ability to provide an electron to the metal surface. The donating procedure gets easier as the **E**_**HOMO**_ becomes higher. Therefore, the ionization potential (**I = - E**_**HOMO**_) should be low. Conversely, the **E**_**LUMO**_ measures the inhibitor molecule’s ability to take an electron from the metal surface; the easier the accepting process, the lower the **E**_**LUMO**_. As a result, the electron affinity (A = - **E**_**LUMO**_) ought to be high. As seen in Fig. 15, a more reactive inhibitor molecule toward the anti-corrosive action is indicated by a HOMO-LUMO energy gap (**ΔE = E**_**LUMO**_**-E**_**HOMO**_) that is as small as possible. All of the parameters have been calculated and recorded in Table 9^31^. High-reactivity compounds that can accept and transmit electrons through metal-inhibitor interaction are known as inhibitors with lower electronegativity, lower hardness, and higher softness. The calculated dipole moments of Co(II), Ni(II), and Cu(II) complexes are 6.969895, 9.677214, and 16.38234 Debye, respectively. The ligand’s value is 4.967052 D. The determined dipole moments of the metal complexes and the ligand indicate that the complexes’ dipole moments are larger than the ligand’s. This phenomenon could be caused by metal ion chelation with the ligand’s active site.

**Table 9:** The calculated quantum chemical parameters for the investigated ligand and its metal complexes.

Quantum chemical parameters	Ligand	Co(II)	Ni(II)	Cu(II)
HOMO (au)	− 0.23909	− 0.22981	− 0.16301	− 0.18798
LUMO (au)	− 0.09164	− 0.12503	− 0.08625	− 0.11960
ΔE	0.14745	0.10478	0.07676	0.06838
I (au)	0.23909	0.22981	0.16301	0.18798
A (au)	0.09164	0.12503	0.08625	0.11960
η (au)	0.073725	0.052390	0.03838	0.03419
s (au^− 1^)	13.56391	19.0876	26.0552	29.24832
ᵡ (au)	0.16536	0.17755	0.12467	0.15379
Chemical potentials µ (au)	− 0.16536	− 0.17755	− 0.12467	− 0.15379
Electrophilicity index ω (au)	0.18152	0.30086	0.20248	0.34588
Reactivity index ΔN (au)	2.242930	3.389006	3.248306	4.498099
Dipole moment (Debye)	4.967052	6.969895	9.677214	16.38234
E (au)	− 1025.8809	− 1353.4232	− 1362.7347	− 1238.8093

#### Molecular electrostatic potential (MEP)

The more helpful descriptor for describing the sites of electrophilic and nucleophilic interactions is the MEP map. The MEP’s three-dimensional view displays the polarized electron density. It provides details about the size and shape of the molecule. On the colored scheme map, the areas with low electron densities are represented by blue parts, while the areas with high electron densities are represented by red or orange sections. The ligand’s O9 and N12 atoms have a (-ve) electrostatic potential, as shown in Fig. 13, which suggests that they can connect to the metal ion coordination center’s (+ ve) electrostatic potential. As a result, the MEP analysis shows that the complexes’ coordination centers have a negative electrostatic potential while their skeletons have a positive electrostatic potential.

## Conclusion

Novel azo metal complexes derived from 5-aminoindazole diazonium salt with benzoyl acetone were successfully synthesized and fully characterized. The spectroscopic and analytical data confirmed the formation of stable 1:1 (M: L) bidentate complexes with coordination through the azo nitrogen and carbonyl groups. The nanocrystalline nature of the compounds was established. The synthesized ligand and its Co(II), Ni(II), and Cu(II) complexes demonstrated promising potential as atmospheric corrosion inhibitors of iron steel, significantly reducing the corrosion rates compared to untreated specimens. Among the tested compounds, the Cu(II) complex exhibited the highest inhibition efficiency. The tetrahedral geometry of the Cu(II) complex contributes mechanistically to its enhanced inhibition efficiency for these reasons (i) it reduces the steric hindrance that enables a closer surface approach, (ii) its small HOMO-LUMO gap and the high electrophilicity favor stronger electronic interaction with the Fe(110) surface, (iii) its favorable adsorption geometry (shorter vertical distance and larger planar coverage) maximize the contact with the Fe(110) surface, compared to the bulkier octahedral Co(II) and Ni(II) complexes. The % IE is arranged as Cu(II) complex (94.60%) **>** Ni(II) complex (77.84%) **>** L (77.26%) **>** Co(II) complex (76.68%). Overall, these findings suggest that the Cu(II) complex shows promise for potential industrial applications.

### Sustainability and safety aspects

From a sustainability standpoint, the azo-benzoyl acetone metal complexes developed in this study offer a promising corrosion inhibition. The inhibitors demonstrated effective corrosion protection at relatively low concentrations, reducing the materials consumption and minimizing environmental burden in potential industrial applications. The ease synthesis of these metals under mild reaction conditions contributes to a lower energy footprint compared to more energy-intensive inhibitor production routes. Among the three metal complexes investigated, the copper complex exhibited the highest corrosion inhibition efficiency and the most favorable safety and environmental profile. Copper is relatively nontoxic to humans and is even recognized as an essential trace element in biological systems.

### Prospective study

The present study provides information on the novel azo β-diketone ligand, its metal complexes, and an evaluation of their corrosion inhibition properties. The study demonstrates their potential value in industrial applications, particularly in reducing atmospheric corrosion and degradation of steel. The thorough investigations indicated that the Cu(II) complex achieved the highest corrosion inhibition efficiency of 94.60%. Therefore, it can be considered that the Cu(II) complex could be a candidate for future industrial applications as an effective and reliable corrosion inhibitor for steel.

## Supplementary Information

Below is the link to the electronic supplementary material.

Supplementary Material 1

## Data Availability

All data generated or analyzed during this study are included in this published article and its supplementary information file.
