# Supplementary material for: Novel azo-benzoyl acetone metal complexes for inhibition of atmospheric steel corrosion
Source: Sci Rep. 2026 Aug 7;16:24531. doi: 10.1038/s41598-026-64011-y (PMC13451279; doi:10.1038/s41598-026-64011-y)
Supplement: Supplementary file 1 [file 41598_2026_64011_MOESM1_ESM.docx]

**Novel Azo-benzoyl acetone metal complexes for inhibition of atmospheric steel corrosion**

Yusif S. El-Sayed^a^, Nadia El-Wakiel^a*^, Kamal El-Baradie^a^, Basma M. Salem^a*^

**^a^**Chemistry Department, Faculty of Science, Tanta University, 31527 Tanta, Egypt

**2.1. Chemicals and Instrumentations**

C, H, and N analyses of the synthesized compounds were carried out using a FLASH 2000 CHNS/O analyzer. The ^1^H-NMR and ^13^C-NMR spectra were recorded on a DELTA2-NMR (500 MHz) in DMSO-d_6_ at 25 c^o^ with tetramethylsilane (TMS) as an internal standard. The FT-IR spectra were taken on an FT-IR-4100 (JASCO, Japan) spectrophotometer in the range of 4000-400 cm^-1^. The standard electron impact mass spectra of the ligand and metal chelates were performed at room temperature, utilizing the mass analyzer's direct inlet component in the thermal scientific GCMS model ISQ. The direct insertion probe (DIP) was gradually heated from 50 to 3000 c^o^ while the ion source was maintained at 25 c^o^. The molar conductance in 10^-3^ M DMSO was calculated. The electronic absorption spectra were obtained using a Shimadzu UV-Vis 240 spectrophotometer. The X-ray diffractometer (GNR, APD 2000 PRO, Italy) was employed to have the XRD patterns of the solid compounds. The magnetic susceptibility of the chelates was carried out at 25 c^o^ using the magnetic susceptibility balance Johnson Matthey 436 Devon Park Drive by Gouy's method [31]. The Shimadzu TG-50 thermogravimetric analyzer was used to perform the thermogravimetric analysis (TGA) of the solid chelates in the range of 25–800 c^o^, at a heating rate of 10 c^o^ /min in an N_2_ atmosphere. An Inductive Coupled Plasma spectrometer (Optima 7000 DV, ICP-OES) was used to measure and identify the elements within the chelate samples.

**Figure S1.** EI‐Mass spectrum of Co (II) complex

**Figure S2**. EI‐Mass spectrum of Ni (II) complex

**Figure S3**. EI‐Mass spectrum of Cu (II) complex

**Table S1.** Bond lengths, and bond angles of Azo-dye ligand

| **Bond Length (^o^A)** | | **Bond angles (^o^)** | |
| --- | --- | --- | --- |
| C1-C2 | 1.396 | C4-C5-C7 | 123.5 |
| C2-C3 | 1.393 | C3-C4-C5 | 120.3 |
| C3-C4 | 1.392 | C5-C7-O9 | 121.6 |
| C4-C5 | 1.400 | C5-C7-C8 | 120.9 |
| C5-C6 | 1.402 | C1-C6-C5 | 120.5 |
| C5-C7 | 1.494 | C7-C8-C10 | 115.5 |
| C7-C8 | 1.549 | C7-C8-N23 | 111.9 |
| C7-O9 | 1.212 | C8-C10-C11 | 116.4 |
| C8-C10 | 1.549 | C8-C10-O22 | 120.4 |
| C10-O22 | 1.206 | C11-C10-O22 | 123.2 |
| C10-C11 | 1.511 | C6- C5-C7 | 117.4 |
| C8-N23 | 1.464 | C10-C8-N23 | 111.9 |
| N23-N12 | 1.248 | C8-N23-N12 | 112.4 |
| N12-C13 | 1.421 | N23-N12-C13 | 116 |
| C13-C18 | 1.392 | N12-C13-C14 | 114.5 |
| C13-C14 | 1.417 | N12-C13-C18 | 124.4 |
| C18-C17 | 1.396 | C15-C16-N19 | 132.4 |
| C14-C15 | 1.380 | C16-N19-N20 | 112.7 |
| C15-C16 | 1.403 | N19-N20-C21 | 106 |
| C16-C17 | 1.421 | N20-C21-C17 | 111.5 |
| C16-N19 | 1.421 | C21-C17-C18 | 135.8 |
| N19-N20 | 1.361 | C16-C17-C18 | 120 |
| N20-C21 | 1.315 | C16-C17-C21 | 104.2 |
| C21-C17 | 1.432 | C17-C18-C13 | 118 |

**Table S2.** Bond lengths, and bond angles of Co (II) complex

| **Bond Length (^o^A)** | | **Bond angles (^o^)** | |
| --- | --- | --- | --- |
| Co-N14 | 2.066 | O8-Co-O25 | 167.5 |
| Co-O25 | 2.290 | Cl28-Co-N14 | 168.6 |
| Co-Cl28 | 2.444 | Cl27-Co-O26 | 174.9 |
| Co-O8 | 2.137 | O8-Co-Cl28 | 91.8 |
| Co-Cl27 | 2.331 | Cl28-Co-O25 | 78.80 |
| Co-O26 | 2.071 | O25-Co-N14 | 106.18 |
| O8-C7 | 1.257 | N14-Co-O8 | 84.60 |
| C7-C3 | 1.482 | Cl28-Co-O26 | 78.20 |
| C3-C2 | 1.417 | O26-Co-O25 | 91.81 |
| C3-C4 | 1.421 | N14-Co-Cl27 | 90.52 |
| C4-C5 | 1.401 | Cl27-Co-O8 | 90.68 |
| C7-C9 | 1.563 | Co-O8-O7 | 127.39 |
| C9-C10 | 1.539 | O8-C7-C9 | 113.98 |
| C10-O11 | 1.248 | C7-C9-N13 | 109.69 |
| C10-C12 | 1.513 | C9-N13-N14 | 118.67 |
| C9-N13 | 1.496 | N13-N14-Co | 124.03 |
| N13-N14 | 1.285 | N13-N14-C15 | 113.59 |
| N14-C15 | 1.446 | N14-C15-C20 | 117.30 |
| C15-C16 | 1.403 | N14-C15-C16 | 120.77 |
| C15-C20 | 1.427 | C18-N23-N22 | 112.36 |
| C18-N23 | 1.380 | N23-N22-C21 | 105.60 |
| N23-N22 | 1.395 | O8-C7-C3 | 119.45 |
| N22-C21 | 1.342 | C7-C3-C4 | 116.12 |
| C21-C17 | 1.442 | C7-C3-C2 | 124.30 |
| C17-C16 | 1.406 | C3-C2-C1 | 119.84 |
| C20-C19 | 1.394 | C3-C4-C5 | 120.23 |
| C19-C18 | 1.413 | C15-C20-C19 | 120.82 |
| C18-C17 | 1.433 | C15-C16-C17 | 117.86 |

**Table S3.** Bond lengths, and bond angles of Ni (II) complex

| **Bond Length (^o^A)** | | **Bond angles (^o^)** | |
| --- | --- | --- | --- |
| Ni-N14 | 2.035 | N14-Ni-O25 | 168.30 |
| Ni-O25 | 1.949 | O26-Ni-Cl28 | 164.88 |
| Ni-O26 | 2.003 | O8-Ni-Cl27 | 83.22 |
| Ni-O8 | 1.947 | Cl27-Ni-O26 | 46.58 |
| Ni-Cl28 | 2.452 | O26-Ni-N14 | 93.67 |
| Ni-Cl27 | 3.932 | N14-Ni-Cl28 | 90.21 |
| O8-C7 | 1.279 | Cl28-Ni-O8 | 89.59 |
| C7-C3 | 1.514 | Cl27-Ni-O25 | 47.11 |
| C3-C2 | 1.422 | Ni-O8-C7 | 127.33 |
| C3-C4 | 1.423 | O8-C7-C9 | 124.15 |
| C4-C5 | 1.405 | C7-C9-N13 | 123.19 |
| C7-C9 | 1.569 | C9-N13-N14 | 115.53 |
| C9-C10 | 1.629 | N13-N14-Ni | 132.62 |
| C10-O11 | 1.247 | N13-N14-C15 | 111.49 |
| C10-C12 | 1.554 | N14-C15-C20 | 117.19 |
| C9-N13 | 1.537 | N14-C15-C16 | 120.84 |
| N13-N14 | 1.331 | C18-N23-N22 | 113.16 |
| N14-C15 | 1.486 | N14-N22-C21 | 102.93 |
| C15-C16 | 1.412 | O8-C7-C3 | 118.05 |
| C15-C20 | 1.440 | C7-C3-C4 | 117.29 |
| C18-N23 | 1.400 | C7-C3-C2 | 122.86 |
| N23-N22 | 1.442 | C3-C2-C1 | 119.60 |
| N22-C21 | 1.370 | C3-C4-C5 | 120.07 |
| C21-C17 | 1.451 | C15-C20-C19 | 120.96 |
| C17-C16 | 1.416 | C15-C16-C17 | 117.90 |
| C20-C19 | 1.394 | C16-C17-C18 | 119.52 |
| C19-C18 | 1.426 | C16-C17-C21 | 135.12 |
| C18-C17 | 1.441 | C20-C19-C18 | 117.21 |

**Table S4.** Bond lengths, and bond angles of Cu (II) complex

| **Bond Length (^o^A)** | | **Bond angles (^o^)** | |
| --- | --- | --- | --- |
| Cu-N12 | 2.142 | O9-Cu-Cl25 | 120.12 |
| Cu-O9 | 2.260 | Cl25-Cu-Cl26 | 94.05 |
| Cu-Cl25 | 2.359 | Cl26-Cu-N12 | 97.63 |
| Cu-Cl26 | 2.419 | N12-Cu-O9 | 85.93 |
| O9-C7 | 1.264 | Cu-O9-C7 | 118.84 |
| C7-C8 | 1.587 | O9-C7-C5 | 120.79 |
| C8-C10 | 1.576 | O9-C7-C8 | 118.84 |
| C10-C11 | 1.553 | C7-C8-C10 | 109.39 |
| C10-O22 | 1.262 | C7-C8-N23 | 110.11 |
| C8-N23 | 1.558 | N23-C8-C10 | 110.70 |
| N23-N12 | 1.336 | C8- C10-O22 | 120.97 |
| C7-C5 | 1.520 | C8-C10-C11 | 117.05 |
| C5-C4 | 1.419 | C7-C5-C6 | 116.17 |
| C5-C6 | 1.422 | C7-C5-C4 | 124.29 |
| C4-C3 | 1.407 | C8-N23-N12 | 110.46 |
| N12-C13 | 1.473 | N23-N12-C13 | 112.55 |
| C13-C18 | 1.413 | N23-N12-Cu | 127.69 |
| C18-C17 | 1.416 | N12-C13-C18 | 116.81 |
| C13-C14 | 1.444 | N12-C13-C14 | 121.28 |
| C14-C15 | 1.390 | C14-C15-C16 | 117.26 |
| C15-N16 | 1.429 | C16-N19-N20 | 113.19 |
| N19-N20 | 1.445 | N19-N20-C21 | 102.84 |
| N20-C21 | 1.370 | N20-C21-C17 | 112.99 |
| C16-N19 | 1.398 | C13-C18-C17 | 117.84 |
| C16-C17 | 1.440 | C13-C14-C15 | 120.96 |

**Table S5.** The Milliken charge distribution for the ligand and its metal complexes

| **Ligand** | | **Co (II) complex** | | **Ni (II) complex** | | **Cu (II) complex** | |
| --- | --- | --- | --- | --- | --- | --- | --- |
| **Atom** | **Charge** | **Atom** | **Charge** | **Atom** | **Charge** | **Atom** | **Charge** |
| C1 | -0.094 | C1 | -0.241 | C1 | -0.069 | C1 | -0.069 |
| C2 | -0.059 | C2 | -0.330 | C2 | -0.070 | C2 | -0.060 |
| C3 | -0.111 | C3 | 0.278 | C3 | -0.015 | C3 | -0.072 |
| C4 | -0.02 | C4 | -0.313 | C4 | -0.049 | C4 | -0.068 |
| C5 | -0.142 | C5 | -0.226 | C5 | -0.067 | C5 | -0.018 |
| C6 | -0.017 | C6 | -0.205 | C6 | -0.056 | C6 | -0.056 |
| C7 | 0.294 | C7 | 0.251 | C7 | 0.201 | C7 | 0.177 |
| C8 | -0.156 | O8 | -0.244 | O8 | -0.103 | C8 | -0.043 |
| O9 | -0.234 | C9 | -0.297 | C9 | -0.034 | O9 | -0.103 |
| C10 | 0.214 | C10 | 0.190 | C10 | 0.181 | C10 | 0.197 |
| C11 | -0.303 | O11 | -0.237 | O11 | -0.140 | C11 | -0.235 |
| N12 | -0.175 | C12 | -0.706 | C12 | -0.241 | N12 | -0.090 |
| C13 | 0.001 | N13 | -0.072 | N13 | -0.061 | C13 | 0.074 |
| C14 | -0.061 | N14 | -0.198 | N14 | -0.091 | C14 | -0.063 |
| C15 | -0.037 | C15 | 0.429 | C15 | 0.071 | C 15 | -0.082 |
| C16 | 0.310 | C16 | -0.439 | C16 | -0.063 | C16 | 0.093 |
| C17 | -0.298 | C17 | 0.239 | C17 | -0.019 | C17 | -0.913 |
| C18 | 0.029 | C18 | 0.271 | C18 | 0.092 | C18 | -0.039 |
| N19 | -0.358 | C 19 | -0.349 | C 19 | -0.078 | N19 | -0.201 |
| N20 | -0.164 | C20 | -0.309 | C20 | -0.053 | N20 | -0.116 |
| C21 | 0.137 | C21 | -0.435 | C21 | -0.018 | C21 | -0.009 |
| O22 | -0.274 | N22 | -0.005 | N22 | -0.116 | O22 | -0.091 |
| N23 | -0.175 | N23 | -0.423 | N23 | -0.201 | N23 | -0.069 |
| H24 | 0.101 | Co24 | 0.078 | Ni24 | 0.148 | Cu24 | 0.197 |
| H25 | 0.100 | O25 | -0.693 | O25 | -0.272 | Cl25 | -0.432 |
| H26 | 0.096 | O26 | -0.684 | O26 | -0.276 | Cl26 | -0.428 |
| H27 | 0.105 | Cl27 | -0.352 | Cl27 | -0.700 | H27 | 0.098 |
| H28 | 0.113 | Cl28 | -0.402 | Cl28 | -0.562 | H28 | 0.095 |
| H29 | 0.164 | H29 | 0.235 | H29 | 0.095 | H29 | 0.092 |
| H30 | 0.134 | H30 | 0.270 | H30 | 0.098 | H30 | 0.084 |
| H31 | 0.137 | H31 | 0.287 | H31 | 0.117 | H31 | 0.115 |
| H32 | 0.137 | H32 | 0.239 | H32 | 0.100 | H32 | 0.107 |
| H33 | 0.109 | H33 | 0.235 | H33 | 0.097 | H33 | 0.106 |
| H34 | 0.100 | H34 | 0.357 | H34 | 0.127 | H34 | 0.116 |
| H35 | 0.115 | H35 | 0.237 | H35 | 0.098 | H35 | 0.107 |
| H36 | 0.243 | H36 | 0.239 | H36 | 0.108 | H36 | 0.107 |
|  |  | H37 | 0.253 | H37 | 0.109 | H37 | 0.095 |
|  |  | H38 | 0.260 | H38 | 0.109 | H38 | 0.134 |
|  |  | H39 | 0.244 | H39 | 0.104 | H39 | 0.247 |
|  |  | H40 | 0.321 | H40 | 0.103 | H40 | 0.115 |
|  |  | H41 | 0.271 | H41 | 0.105 |  |  |
|  |  | H42 | 0.334 | H42 | 0.250 |  |  |
|  |  | H43 | 0.414 | H43 | 0.254 |  |  |
